# Assessing Challenges of 2D-Molybdenum Ditelluride for Efficient Hydrogen Generation in a Full-Scale Proton Exchange Membrane (PEM) Water Electrolyzer

**DOI:** 10.1021/acssuschemeng.3c06616

**Published:** 2024-01-06

**Authors:** Arun Kumar Samuel, Abdulhai H. Faqeeh, Weihao Li, Zeliha Ertekin, Yuanshen Wang, Jingyi Zhang, Nikolaj Gadegaard, David A. J. Moran, Mark D. Symes, Alexey Y. Ganin

**Affiliations:** † School of Chemistry, University of Glasgow, Glasgow G12 8QQ, U.K.; ‡ Department of Chemistry, King Khalid University, Guraiger, Abha 62529, Saudi Arabia; § School of Engineering, University of Glasgow, Glasgow G12 8LT, U.K.

**Keywords:** hydrogen production by water splitting, chemical vapor deposition, proton exchange membrane electrolyzer, 2D materials, transition-metal dichalcogenides

## Abstract

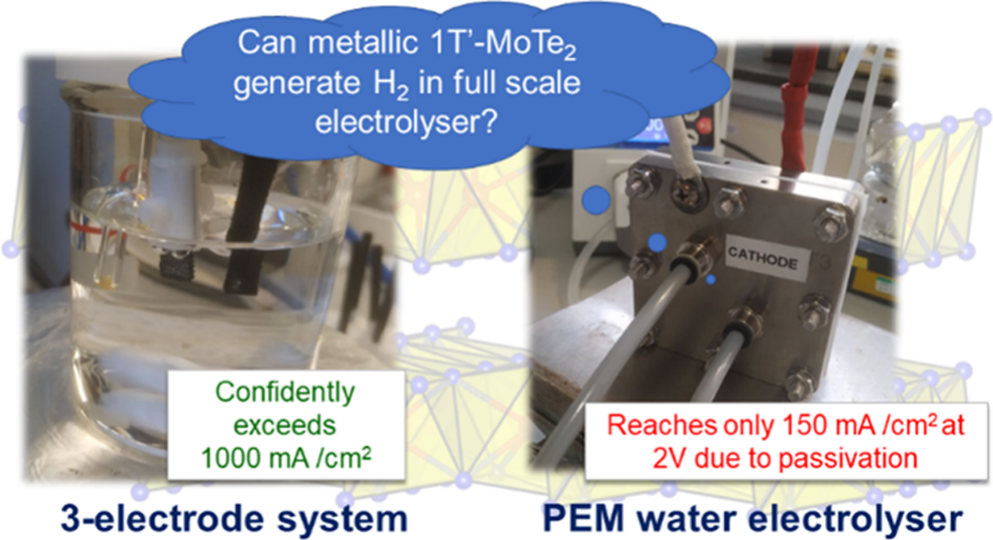

Proton exchange membrane (PEM) water electrolyzers are critical enablers for sustainable green hydrogen production due to their high efficiency. However, nonplatinum catalysts are rarely evaluated under actual electrolyzer operating conditions, limiting knowledge of their feasibility for H_2_ production at scale. In this work, metallic 1T′-MoTe_2_ films were synthesized on carbon cloth supports via chemical vapor deposition and tested as cathodes in PEM electrolysis. Initial three-electrode tests revealed that at 100 mA cm^–2^, the overpotential of 1T′-MoTe_2_ approached that of leading 1T′-MoS_2_ systems, confirming its promise as a hydrogen evolution catalyst. However, when tested in a full-scale PEM electrolyzer, 1T′-MoTe_2_ delivered only 150 mA cm^–2^ at 2 V, far below expectations. Postelectrolysis analysis revealed an unexpected passivating tellurium layer, likely inhibiting catalytic sites. While initially promising, the unanticipated passivation caused 1T′-MoTe_2_ to underperform in practice. This highlights the critical need to evaluate emerging electrolyzer catalysts in PEM electrolyzers, revealing limitations of the idealized three-electrode configuration. Moving forward, validation of model systems in actual electrolyzers will be key to identifying robust nonplatinum catalysts for sustainable green hydrogen production.

## Abstract 2

Assessing metallic 2D chalcogenide in a full-scale water electrolyzer reveals obstacles to designing sustainable, earth-abundant catalysts replacing platinum

## Introduction

Water electrolysis presents one of the most promising opportunities for generating green hydrogen, particularly when the process is powered by renewable energy sources during times of excess production.^1−4^ In this context, proton exchange membrane (PEM) electrolyzers have emerged as critical enablers for hydrogen production due to their rapid start-up capabilities and high efficiency.^5−8^ Since electricity costs dominate over capital expenses (as a typical electrolyzer operates for over 10,000 h), the choice of the catalyst that drives the hydrogen evolution reaction (HER) forward is important.^9,10^ The electrolysis catalyst must be durable because HER occurs near the PEM membrane, which creates highly acidic conditions. Furthermore, as the rate of the HER increases with temperature, the catalyst must be capable of withstanding high cathodic current densities at low voltages and remain operational above 60 °C. High electrical conductivity of the system is vital to minimizing ohmic losses and further improving the H_2_ output efficiency as well. Platinum meets these stringent requirements and remains operational under high reductive currents, low pH, and elevated temperatures over thousands of hours of continuous operation. Substantial reduction of Pt loading without affecting electrolyzer performance is possible, but with it being scarce and expensive, searching for alternatives is still important.^11−14^

Among the many alternatives to Pt being explored, two-dimensional transition-metal dichalcogenides (TMDCs) with metallic conductivity have attracted much interest.^15−18^ Two binary molybdenum-based materials (specifically, metallic 1T′-MoS_2_ and 1T′-MoTe_2_) consistently outperform other TMDCs, and thus, they are probably the most promising targets for testing at scale as Pt alternatives.^19−23^ However, many previous studies are too fundamental in nature: most TMDC catalysts are tested under conditions far removed from those found in actual PEM electrolyzers. For example, the catalytic properties of TMDCs are traditionally tested in trivial three-electrode (half-cell) configurations at extremely low current densities of <10 mA cm^–2^. This pales in comparison to the minimum 500 mA cm^–2^ required in real PEM electrolyzers.^7,13,24,25^ Furthermore, a typical lab-scale procedure is to test for the HER performance in acids such as H_2_SO_4_ or HClO_4_. In contrast, PEM electrolyzers operate with a constant flow of highly purified water, while the acidity is provided by the Nafion membrane. Therefore, more rigorous critical evaluations of TMDCs are needed under actual PEM electrolysis operating conditions if they are ever to be proven as viable alternatives to Pt.^24,25^ As mentioned above, among Mo-based TMDCs, 1T′-MoS_2_ and 1T′-MoTe_2_ are the most promising binary compounds. However, to the best of our knowledge, only the metallic 1T′-MoS_2_ has been tested in a full-scale PEM electrolyzer,^26,27^ while the analogous experimental data for 1T′-MoTe_2_ are still lacking.

Although 1T′-MoTe_2_ has never been tested in an electrolyzer, there are numerous reports suggesting it is more than capable of sustaining high current densities in sulfuric acid.^23,28−31^ A recent report has suggested that it has a lower overpotential than Pt at a given current density, however, only when tested in a three-electrode (half-cell) setup in sulfuric acid.^31^ The main attraction of using 1T′-MoTe_2_ is that it can be grown as ultrathin films directly on common gas-diffusion layers (GDLs) such as carbon cloth (CC), thus circumventing the need for immobilizing it on a Nafion membrane or on a GDL by spraying. The direct film growth can minimize the deformation (and cracking) of the catalyst layer at the membrane assembly interface and lead to improved durability of the electrolyzer.^32^ Moreover, thin-film growth technology is a highly automated process, and thus, it could warrant the quality and reliability of electrolyzer devices based on metallic 1T′-MoTe_2_ thin films compared with sprayed interfaces. Recent successes in fabrication of 1T′-MoTe_2_ films on CC^33,34^ demonstrated that large film areas (imperative for electrolyzer applications) are achievable. This enables a pivotal shift to rigorous evaluation of the films in industrially relevant full-scale flow-cell PEM electrolyzers. In turn, evaluating the applicability of this two-dimensional (2D) material as catalyst for water electrolysis would signify a crucial evolutionary step in the field, one that is long overdue and necessitates prioritization moving ahead.

In this work, we leverage our expertise with the MoTe_2_ system to answer the question whether metallic 1T′-MoTe_2_ is a viable alternative to 1T′-MoS_2_ and Pt catalysts. We pioneered a synthetic approach to leverage carbon cloth as both a gas-diffusion layer and a catalyst support, accomplishing the growth of metallic 1T′-MoTe_2_ films directly on carbon cloth using a combination of electrodeposition and chemical vapor deposition (CVD). We also identified growth conditions for 2H- and 1T′-/2H-MoTe_2_ films on CC, which were then tested in a three-electrode system (H_2_SO_4_ electrolyte) and compared to 1T′-MoTe_2_ films. The three-electrode system results confirmed that 1T′-MoTe_2_ was seemingly an excellent catalyst for the HER, and therefore, the final part of this research was focused solely on 1T′-MoTe_2_ films. When tested in a PEM electrolyzer, 1T′-MoTe_2_/CC achieved the current density of 150 mA cm^–2^ at a 2 V cell potential. However, this performance was inferior to that demonstrated by Pt, which gave current densities of >1000 mA cm^–2^ at 2 V. The postelectrolysis characterization experiments suggested that the formation of a thin layer of Te at the surface of 1T′-MoTe_2_/CC prevented it from achieving a good performance. In this context, 1T′-MoS_2_ may in fact be a better alternative to Pt as it does not seem to suffer from this surface passivation drawback and tends to achieve significantly higher current densities at a given potential.

## Experimental Section

### Electrodeposition of MoO_3_ and Chemical Vapor Deposition of MoTe_2_ Films on Carbon Cloth Support

For the preparation of the electrolyte, 5 mM (NH_4_)_6_Mo_7_O_24_·4H_2_O (Alfa Aesar, 99%) and 5 mL of HCl (Sigma-Aldrich, 38%) were dissolved in 100 mL of deionized water over 30 min. Carbon cloth (W0S1011, Fuel Cell Store) was used as a working electrode. The electrodeposition was carried out in a Biologic (SP-150) electrochemical workstation at an optimized potential of −0.8 V vs Ag/AgCl to attain uniform deposition. Carbon cloth was used as the working electrode, Ag/AgCl (3 M NaCl saturated, CHI Instruments) as the reference electrode, and carbon-fiber felt (Alfa Aesar, 99%) as the counter electrode. After 30 min of electrodeposition, the carbon cloth was washed with deionized water, dried at 60 °C for 30 min, and heated at 450 °C for 3 h. To prepare MoTe_2_, the electrodeposited MoO_3_ on the carbon cloth sample was placed in a CVD reactor (see Supporting Note 1 for details). The conversion to MoTe_2_ was carried out at a heating rate maintained at 5 °C min^–1^ with a dwell time of 4 h, before cooling to room temperature at 5 °C min^–1^. The carrier gas was a mixture of 5% H_2_ in Ar (BOC) at a flow rate of ∼125 sccm. A detailed description of the electrodeposition of MoO_3_ and CVD of MoTe_2_ is given in Supporting Note 1.

### Electrochemical Measurements in a Three-Electrode Configuration

Electrochemical experiments in a three-electrode (half-cell) configuration were performed on a Biologic SP-150 potentiostat (EC Laboratories) using MoTe_2_ on carbon cloth as the working electrode, 3 M Ag/AgCl as the reference electrode, and carbon-fiber felt as the counter electrode. Aqueous 1 M H_2_SO_4_ was used as an electrolyte. A detailed description of the experiment is given in Supporting Note 1.

### Fabrication of the RuO_2_/TiO_2_ Microporous Layer on Ti-Fiber Felt

Ti-fiber felt (2.3 × 2.3 cm^2^, 53–56% porosity, thickness 0.25 ± 0.05 mm, Fuel Cell Store) was sprayed with Ti-particles (loading mass of ∼1.8 mg cm^–2^) using an AB-182 double-action suction-feed airbrush (0.5 mm nozzle, Everything Airbrush, U.K.) to form a microporous layer (MPL). The preparation of MPL has been described in detail in Supporting Note 1. Subsequently, the Ti-fiber felt coated with the Ti-microporous layer was sprayed with the RuO_2_ ink with the loading mass of ∼1.8 mg cm^–2^. The ink was prepared by mixing Nafion solution (5 wt %, Sigma-Aldrich), RuO_2_ powder (99% anhydrous, Thermo Scientific), and carbon black (99.9+%, Thermo Scientific) in a 15:20:65 mass ratio.

### Single-Cell PEM Water Electrolyzer Components

Two individual 5 cm^2^ Ti-flow fields with a serpentine channel (T3, v2.0, Dioxide Materials) were used as cathode and anode plates. On the cathode side, a metallic 1T′-MoTe_2_ film on the CC (2.3 × 2.3 cm^2^) was used as a catalyst and a cathode gas-diffusion electrode. The Ti-fiber felt (2.3 × 2.3 cm^2^) with the RuO_2_/TiO_2_ microporous layer was used as the OER catalyst, and the gas-diffusion electrode was used as an anode. Anode (0.0127 cm) and cathode (0.0254 cm) gaskets (PTFE, Fuel Cell Store) with the size of 2.5 × 2.5 cm^2^ were used. A Nafion membrane (N-117, Fuel Cell Stores) with the size of about 2.4 × 2.4 cm^2^ was used as the proton exchange membrane, and it was pretreated in 1 M H_2_SO_4_ (Fisher Scientific, ∼95%) solution at 80 °C for 1 h. A detailed sequence of the electrolyzer assembly and prior testing is given in Supporting Note 1.

### Material Characterization

X-ray diffraction (XRD) experiments were performed on a Rigaku MiniFlex 6G diffractometer (Cu-Kα1 and -Kα2 source) equipped with a D/teX Ultra detector and operating in Bragg–Brentano geometry. Carbon cloth substrates were carefully mounted on a silicon zero-background holder using double-sided tape. Rigaku SmartLab Studio-II software (Rigaku Corporation, 2014) was used for data collection. Raman experiments were carried out on a Horiba Jobin-Yvon LabRam HR-800 spectrometer integrated with a 532 nm laser. Low laser power (25 mW) setting was used to prevent samples from laser-induced thermal degradation. The aperture size was 100 μm, and the spectra were collected with a 10 s accumulation time over 10 repetitions. Optical images were also acquired using a 50× objective lens integrated with the spectrometer. Scanning electron microscopy (SEM) images were attained using a TESCAN CLARA instrument coupled with an Oxford Instruments UltimMax 65 with an Aztec live interface for energy-dispersive X-ray spectroscopy (EDXS) measurements. The MoTe_2_ on carbon cloth was placed over the carbon-adhesive disc (Agar Scientific). The EDX spectrum was initially performed with Cu-foil as the calibration standard. The data sets were processed using the Aztec EDX software. Inductively coupled plasma optical emission spectroscopy (ICP-OES) was performed using an Agilent 5900 ICP-OES, equipped with an Agilent SPS4 autosampler. The instrument was calibrated with reference standards of Mo and Te. Gas chromatography (GC) measurements were performed in an Agilent 8860 Gas Chromatograph system with a thermal conductivity detector. The GC system was calibrated using certified standards of gas mixtures (CK Gas Product Limited, U.K.) before use.

## Results and Discussion

### Electrodeposition of MoO_3_ Films (MoO_3_/CC) and Synthesis of MoTe_2_ (MoTe_2_/CC) on Carbon Cloth

**Figure 1:**
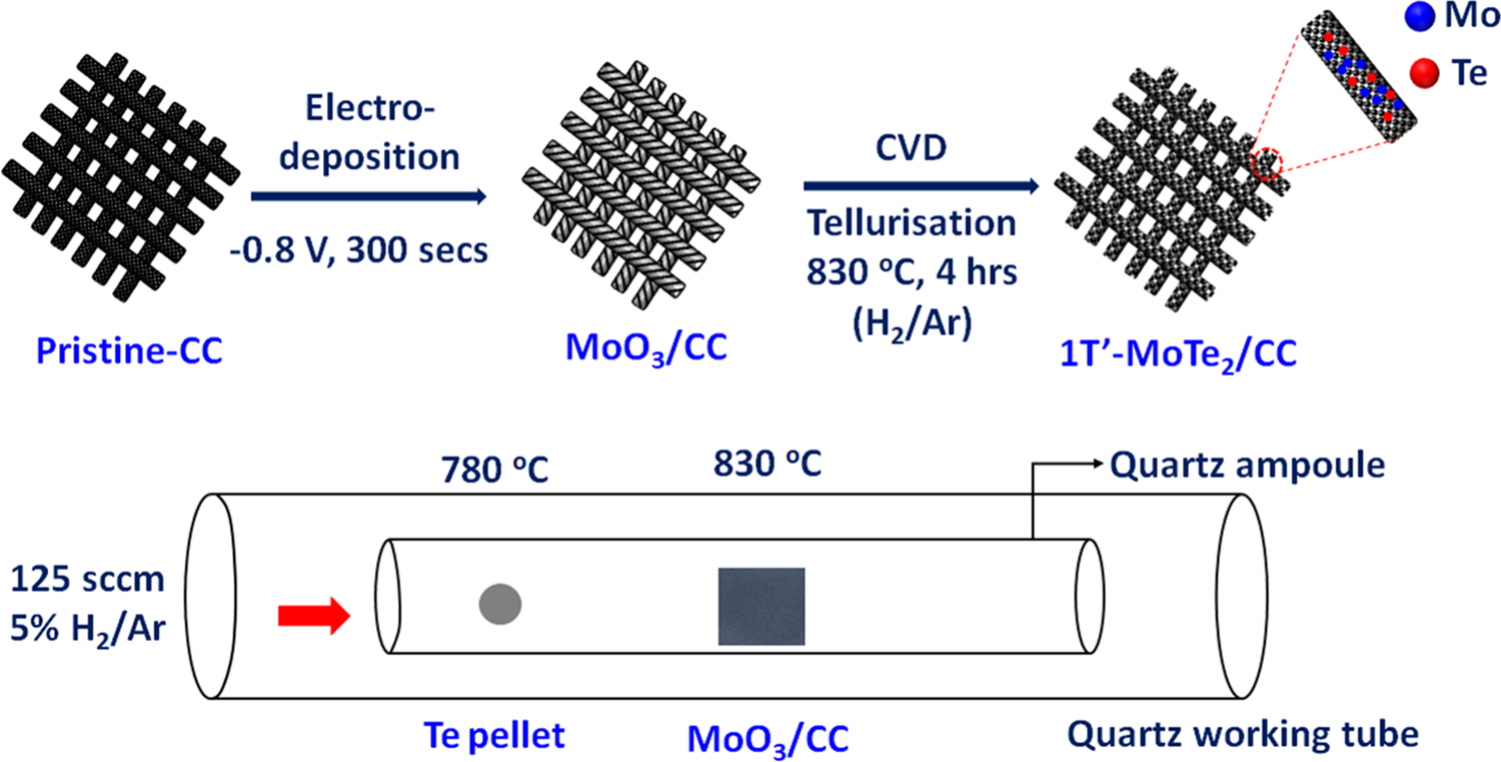
Schematic illustration of the preparation of the metallic 1T′-MoTe_2_ film from electrodeposited α-MoO_3_ on carbon cloth (CC) and the CVD reactor setup.

The growth of metallic 1T′-MoTe_2_ films requires a seeding layer of MoO_3_.^35^ Optimization of a previously reported^36^ electrodeposition process of MoO_3_ on carbon cloth (CC), followed by heating the deposits to 450 °C in air (Figure 1), yields a range of MoO_3_ products depending on the applied potential (Figure S1).

**Figure 2:**
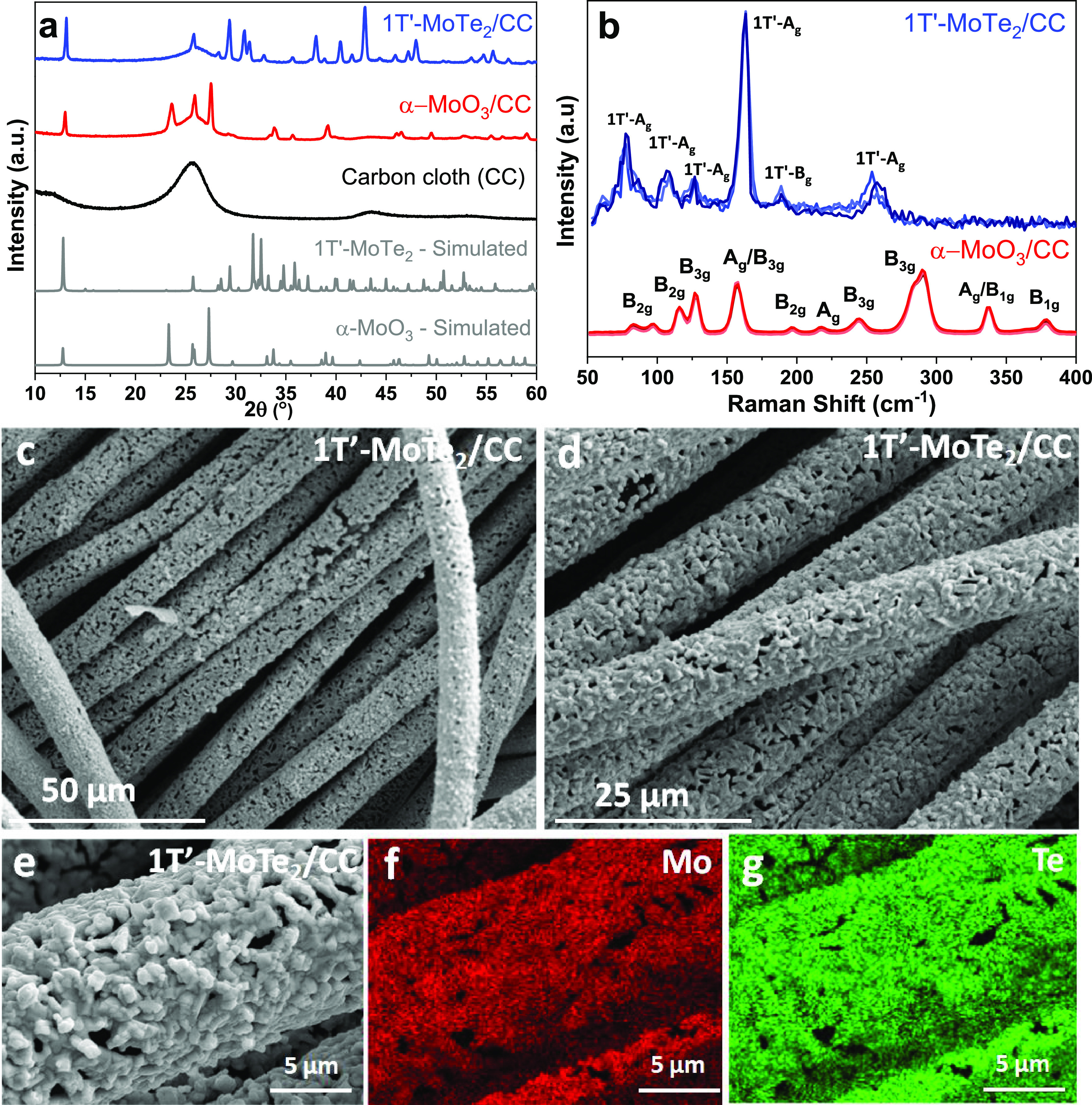
(a) XRD patterns of the electrodeposited MoO_3_ and 1T′-MoTe_2_ grown on the CC support compared with the standard ICSD data of α-MoO_3_ and 1T′-MoTe_2_. (b) Raman spectra of the electrodeposited MoO_3_ and 1T′-MoTe_2_ grown on the CC support. The spectra were recorded at three distinct spots on the individual substrates to show their surface uniformity. (c, d) SEM images of 1T′-MoTe_2_ grown on CC at two different magnifications. (e–g) Higher-resolution SEM and EDX elemental mapping of Mo and Te elements on the 1T′-MoTe_2_ film grown on the CC support.

The optimal MoO_3_ product was obtained by running the electrodeposition for 300 s at −0.8 V as was evidenced by an XRD pattern (Figure 2a) consistent with the one expected for the orthorhombic α-MoO_3_ phase and carbon cloth (Figure S2). Raman spectra further confirmed the formation of MoO_3_ (Figure 2b) as the peak assignment (Table S1) was consistent with the literature.^37^ The almost-perfect overlap of several recorded spectra confirmed that the film thickness of MoO_3_ across CC was homogeneous (Figure S3). The optical images captured at three different regions across the MoO_3_/CC surface showed CC fibers coated in a thin, glossy film: like bracelets enveloping its circumference (Figure S4). Similarly, comparison between scanning electron microscopy (SEM) images recorded on MoO_3_/CC confirmed the formation of a relatively homogeneous film, which is, however, split open periodically (Figure S5). The EDX spectra (Figure S5) showed that the gaps are free from MoO_3_ and are probably just a result of the mechanical damage from deformation rather than the inhomogeneous electrodeposition.

Since XRD, Raman, and SEM/EDX experiments confirmed successful electrodeposition of MoO_3_ on the CC, we proceeded with the synthesis of metallic 1T′-MoTe_2_ films using the CVD apparatus schematically depicted in Figure 1. Our previous work showed that depending on the CVD reaction conditions, MoTe_2_ forms films of either hexagonal, semiconducting (2H-MoTe_2_) or monoclinic, metallic (2H-MoTe_2_) polymorphs as well as mixtures of thereof.^35^ Therefore, a wide range of the reaction parameters (Table S2) was explored in this work to find the reaction conditions. According to XRD, phase-pure 1T′-MoTe_2_/CC films were formed at 830 °C (Figure 2a). These reaction conditions are consistent, for example, with those reported for the 1T′-MoTe_2_ powder^38^ and thin films grown on the SiO_2_/Si-substrate and CC.^33,35,39,40^ Similarly, Raman spectroscopy confirmed the formation of the 1T′-MoTe_2_ phase across the CC substrate (Figure 2b).^41^ The films prepared at lower reaction temperatures gave the diffraction patterns and Raman spectra consistent with mixed 1T′-/2H-MoTe_2_ or 2H-MoTe_2_ films (Figures S6 and S7).

The surface morphology and composition of the key 1T′-MoTe_2_ film on CC were further examined by SEM, revealing that the resulting film covers relatively homogeneously the CC fibers (Figure 2c,d). However, higher magnification images revealed that the film was of a granular nature (Figure 2e). Similar granular films were noticed by previous researchers for the 1T′-MoTe_2_ film on CC.^33^ This is probably caused by the difference in coefficients of thermal expansions between MoTe_2_ and CC. Still, the SEM clearly shows that 1T′-MoTe_2_ films are oriented in plane with the CC substrate. In comparison, the SEM images (Figure S8) recorded on films prepared at lower temperatures consisted of crystallites that were mostly oriented out of plane.

In addition, Te- and Mo-mapping (carried out on a sample displayed in Figure 2e) showed that the elements were evenly distributed within the film (Figure 2f,g), which further suggested the homogeneous nature of the films. EDX analysis (Figure S9) revealed that the stoichiometry of the 1T′-MoTe_2_ films was marginally below the expected 1:2 composition, which is something we noticed on 1T′-MoTe_2_ powders prepared by our group before.^38^ The results of the EDX analysis on other films (such as 2H- and mixed 2H/1T′-samples) are summarized in Table S3.

The difference between the weight of pristine carbon cloth and the sample of 1T′-MoTe_2_/CC gave the loading of 1T′-MoTe_2_ of 0.6 mg cm^–2^. Subsequently, attempts were made to increase the loading by increasing the thickness of MoO_3_ during the electrodeposition process. However, the use of thicker MoO_3_ films invariably led to the formation of films comprising both 1T′- and 2H-MoTe_2_, instead of the desired pure 1T′-phase films (Figure S10).

### Electrochemical Properties of MoTe_2_ Films on Carbon Cloth in H_2_SO_4_ in a Three-Electrode Configuration

**Figure 3:**
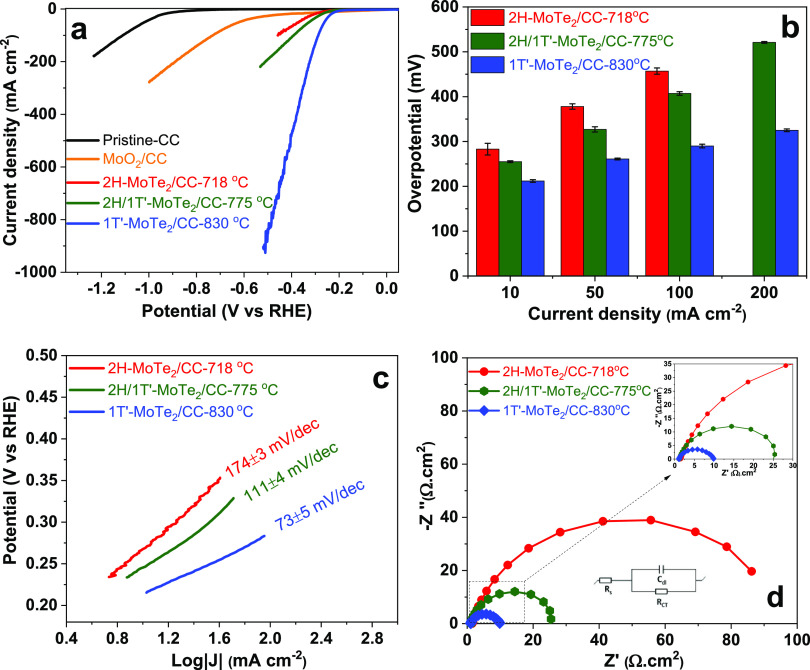
Electrochemical data on MoTe_2_/CC films in a three-electrode configuration in 1 M H_2_SO_4_. (a) LSV polarization curves compared with pristine-CC and MoO_2_/CC. (b) Comparison of overpotential measured at different current densities (10, 50, 100, and 200 mA cm^–2^). (c) Tafel plots and equivalent Tafel slope values extracted from LSV polarization data at different overpotential ranges. (d) Nyquist plots. The inset shows a zoom-in region.

Testing in a PEM electrolysis flow-cell is both time-consuming and expensive, requiring substantial effort, and is therefore impractical for poorly performing catalysts. Therefore, the comparative ability of 2H-, 2H/1T′-, and 1T′-MoTe_2_ films on CC (all with ∼0.6 mg cm^–2^ loading) to evolve hydrogen was initially evaluated by measuring linear sweep voltammetry (LSV) polarization curves in 1 M H_2_SO_4_ in a three-electrode system using Ag/AgCl as a reference electrode and carbon-fiber felt as a counter electrode. The aim of this study was to identify the best-performing MoTe_2_ films (in terms of the current densities at 100 mA cm^–2^) which could then be used in a full-scale single-cell PEM electrolyzer. The polarization curves are shown in Figure 3a, and the overpotential values at selected current densities are summarized in Figure 3b and Table S4. The magnified range including LSV curves within a lower current density is also shown in Figure S11.

The 2H-MoTe_2_/CC film showed low current densities even at high potential values (Figure 3a), consistent with previous reports on the poor catalytic properties of 2H-MoTe_2_.^21,38,23^ However, 2H-MoTe_2_/CC still outperformed CC and MoO_2_/CC controls in terms of overpotential values at given current densities (Figure 3a). The mixed 2H/1T′-MoTe_2_ films showed only minor improvements in performance in comparison with 2H-MoTe_2_ films (Figure 3a), which was consistent with the previously reported work for MoTe_2_ films on CC.^33^ In comparison with 2H- and mixed 2H/1T′-MoTe_2_, the phase-pure 1T′-MoTe_2_/CC film showed much higher current densities (>500 mA cm^–2^), and a lower overpotential was required to reach specific currents. The better HER performance of 1T′-MoTe_2_ can be explained by the metallic conductivity of this polymorph^19^ and the distortion within the crystal structure upon electron doping, which alters the lattice structure.^21^ The alteration improves the Gibbs free energy for efficient hydrogen surface adsorption. In addition, we could not confirm a recent report suggesting that 2H/1T′-MoTe_2_ films^42^ should demonstrate a better performance than pure 1T′-MoTe_2_ films. The report in question highlighted that the heterophase boundary between 2H- and 1T′-MoTe_2_ contains catalytically active sites due to local charge accumulation at the defects. However, the observed performance of 1T′-MoTe_2_/CC that we found contradicted this report, suggesting that intergrowth between 1T′ and 2H-phases does not lead to improved catalytic performance. A possible explanation for the lack of the effect in our case may be that close-to-monolayer films are required in order to observe it.

Overall, the comparative values of overpotentials across different types of films and a comparison with literature analogues on CC are summarized in Table S5. It is evident that 1T′-MoTe_2_/CC appears to be a viable target since the overpotential required to reach 100 mA cm^–2^ was 290 mV. This is only marginally higher than the 245 mV^26^ and 240 mV^27^ observed at the same current densities on 1T′-MoS_2_ samples, which were first tested in a three-electrode system and then consequently in a PEM electrolyzer.

In addition, the Tafel slopes provide a valuable insight into the operability of the catalyst at high current densities,^43^ and as evident from Figures 3c and S11, the phase-pure 1T′-MoTe_2_/CC film exhibited the lowest Tafel slope value of 73 ± 5 mV dec^–1^ among the tested films. The Tafel slope value was close to the value of 67 mV dec^–1^ observed in 1T′-MoS_2_ previously tested in a PEM electrolyzer.^26^ Similarly, the double-layer capacitance (*C*_dl_) and electrochemical impedance analysis from Nyquist plots (Figure 3d) clearly point out that 1T′-MoTe_2_/CC films are good targets for PEM electrolyzer experiments. The calculated *C*_dl_ and *R*_CT_ values for all films (including 2H- and mixed 2H/1T′-films) are given in Figures S12 and Table S6 for comparison. It is evident that the metallic character of 1T′-MoTe_2_ resulted in efficient charge transfer kinetics, as evidenced by the lower *R*_CT_. However, the *R*_CT_ = 8.75 Ω cm^2^ was higher for 1T′-MoTe_2_/CC than the one reported for 1T′-MoS_2_ (*R*_CT_ = 3.22 Ω cm^2^), suggesting that 1T′-MoS_2_ is more conductive.^26^

Finally, the chronoamperometric (CP) measurements as well as post-CP characterization showed that the current density remained stable over time (Figure S13), consistent with previously reported data on 1T′-MoTe_2_.^23^ Furthermore, the ICP-OES analysis showed that the leaching of Mo and Te was minimal, reaching only 2 and 6 ppm, respectively. Post XRD and Raman characterizations (Figure S14) confirmed the retention of the 1T′-MoTe_2_ phase after 15 h of a CP test. Therefore, based on the electrochemical performance achieved in the three-electrode system (LSV polarization curves, EIS, and chronoamperometry stability results), the 1T′-MoTe_2_/CC films were deemed suitable for testing in a single-cell PEM electrolyzer.

### Application of 1T′-MoTe_2_/CC as a HER Electrocatalyst in a Single-Cell PEM Water Electrolyzer

**Figure 4:**
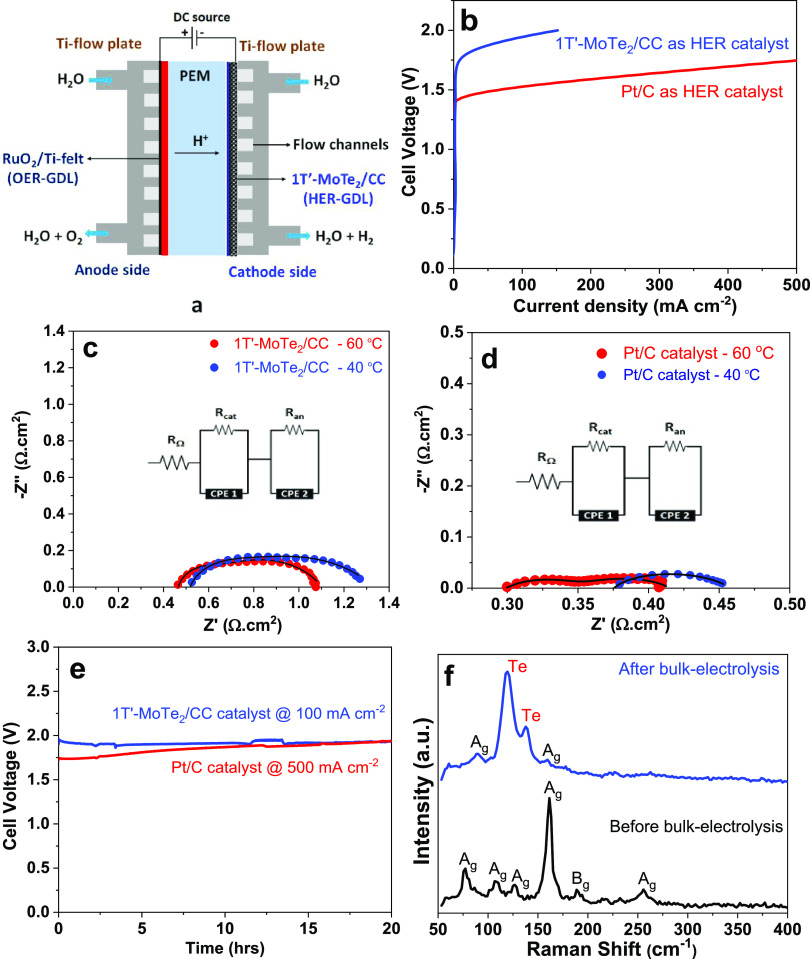
PEM electrolyzer testing with 1T′-MoTe_2_/CC or Pt/C as cathodes and RuO_2_/Ti-fiber felt as the anode. (a) Schematic representation of the PEM electrolyzer used for testing of the electrochemical properties. (b) Comparison of LSV polarization curve of the PEM flow-cell water electrolyzer operated at 60 °C. Nyquist plots of (c) 1T′-MoTe_2_/CC and (d) Pt/C cathodes at 2 V performed at 40 and 60 °C. The respective equivalent circuit used for EIS fitting is also shown. (e) Galvanostatic electrolysis at 60 °C. (f) Raman analysis of the 1T′-MoTe_2_/CC catalyst before and after stability tests in a PEM electrolyzer operated at 60 °C.

To the best of our knowledge, 1T′-MoTe_2_ has never been employed as a HER electrocatalyst in an electrolyzer; therefore, assessing the electrochemical performance of the 1T′-MoTe_2_ films in such a device is crucial. The tests were carried out in a 5 cm^2^ PEM flow-cell water electrolyzer, with the key components schematically shown in Figure 4a, while the overview photographs of the overall assembly are in Figure S15.

Separated by the Nafion 117 membrane, the 2.3 × 2.3 cm^2^ area 1T′-MoTe_2_/CC (0.6 mg_1T′-MoTe_2__ cm^–2^) was used as a cathode and RuO_2_ (1.8 mg_RuO_2__ cm^–2^) immobilized on 2.3 × 2.3 cm^2^ area Ti-fiber felt was used as the anode following an earlier described protocol.^44^ Before applying the voltage, the fully assembled electrolyzer was tested by flowing preheated water at 40 and 60 °C at a flow rate of 18 mL min^–1^. No leakage or any evidence for powder residuals was evident, suggesting that the system was tight and both 1T′-MoTe_2_ HER/RuO_2_ OER electrocatalysts were stable toward delamination. An electrolyzer, consisting of a commercial Pt catalyst on CC (0.5 mg_Pt_ cm^–2^) HER/RuO_2_ OER catalysts, was also assembled and tested under the same conditions, showing no leakages. The choice of Pt catalyst loading at 0.5 mg cm^–2^ was selected based on this being close to the loading of 0.6 mg_1T′-MoTe_2__ cm^–2^ used for the telluride.

The testing of 1T′-MoTe_2_/CC was carried out within a reasonably narrow range (0–2 V) to avoid degradation of RuO_2_, which is prone to oxidation at higher voltage ranges.^45^ The initial LSV testing at 40 °C showed that the maximum current densities of ∼100 mA cm^–2^ were achieved at 2 V (Figure S16). Three subsequent LSV scans revealed that the system was stable, as was evident by the overlap of the recorded curves (Figure S16). Consequently, the operation temperature of the electrolyzer was increased to 60 °C. There was an improvement in charge transfer resistance (*R*_CT_) when the electrolyzer was operated at 60 °C (Figure 4c), which agreed with the LSV result (Figure S17). The current densities achieved at 60 °C on 1T′-MoTe_2_/CC (0.6 mg_1T′-MoTe_2__ cm^–2^) were ∼150 mA cm^–2^: a 50% improvement compared with the values of 100 mA cm^–2^ when the system was operated at 40 °C (Figure S16). However, in comparison, the platinum (0.5 mg_Pt_ cm^–2^) benchmark required a cell potential of just over 1.5 V to achieve the same current densities (150 mA cm^–2^) at 60 °C (Figure 4b). Furthermore, at 2 V, the electrolyzer with the Pt-based cathode achieved a current density of >1000 mA cm^–2^, confirming that at a similar loading of 0.5 mg_Pt_ cm^–2^ on CC, Pt gave a much better result than 1T′-MoTe_2_. The perfect overlap of three recorded LSVs within the 0–2 V range with the Pt/C HER catalyst also confirmed that no degradation of the RuO_2_ OER electrocatalyst took place (Figure S18).

Despite achieving reasonable current densities, the overall performance of 1T′-MoTe_2_/CC is poorer than observed in the recent work on 1T′-MoS_2_ powders.^26,27^ For example, at a low loading (0.4 mg_1T′-MoS_2__ cm^–2^), Garcia et al. reported achieving the current densities (>800 mA cm^–2^) at 2 V; however, it should be noted that the electrolyzer was operated at a higher temperature of 80 °C. Similarly, Xie et al.^27^ achieved current densities >800 mA cm^–2^ at low 1T′-MoS_2_ loading (0.14 mg_1T′-MoS_2__ cm^–2^). Notably, these results are quite different from the experiments in the three-electrode system discussed above, which showed that the difference between 1T′-MoTe_2_ and 1T′-MoS_2_ was minimal. To further understand what might have influenced the performance of 1T′-MoTe_2_/CC films, the determination of charge transfer resistance was carried out. The calculated cathodic resistance value of *R*_cathode_ = 0.15 Ω cm^–2^ (Figure 4c) was marginally greater than that reported for 1T′-MoS_2_ (R_cathode_ = 0.11 Ω cm^–2^).^26^ The charge transfer resistance for a 0.5 mg_Ptcm^–2^_ on CC (*R*_cathode_ = 0.05 Ω cm^–2^) was also calculated and found to be lower (Figure 4d). Therefore, it was unlikely that substantial changes occurred to the cathode that could lead to such a difference in performance (in terms of current densities) between telluride and sulfide.

Galvanostatic electrolysis was employed to test the stability of the 1T′-MoTe_2_/CC cathode at 60 °C under prolonged operational times. The electrolyzer was operated under a constant current density of ∼100 mA cm^–2^ for 20 h to maintain the overall cell potential (Figure 4e). The cell potential remained unchanged for a period of 20 h. However, in comparison, the Pt benchmark required only 1.72 V to operate at a significantly higher current of ∼500 mA cm^–2^ for 20 h with minimal degradation. During the galvanostatic measurements, the H_2_ gas generated on the cathode side was collected through the water displacement technique, as illustrated in Figure S19. Over the course of 1 h, ∼250 mL of hydrogen was collected using this approach, which allowed us to determine the faradic yield for hydrogen production as being 98.6% (Figure S20). The collected gas was also analyzed by gas chromatography (GC), as shown in Figure S20. The chromatogram is dominated by a peak consistent with the presence of hydrogen. Trace amounts of oxygen and nitrogen were detected (due to air leaks during sample collection) as well. These values were at much lower levels than found in a blank Ar-sample, confirming that no oxygen crossover took place between the anode and cathode within the electrolyzer.

To assess the energy efficiency of the electrolysis process, we used the formulas and procedures reported previously.^26^ First, an efficiency value of η = 75.5% at ∼150 mA cm^–2^ was obtained for the 1T′-MoTe_2_/CC (0.6 mg_1T′-MoTe_2__ cm^–2^) catalyst. We also took the value of ∼1.8 V (required to achieve 150 mA cm^–2^) from the previously reported LSV plot for 1T′-MoS_2_ (0.4 mg_1T′-MoTe_2__ cm^–2^)^26^ to obtain a higher η > 80% for this sulfide. However, in comparison with Pt (0.5 mg_Pt_ cm^–2^), which showed an efficiency value of η > 95% at 150 mA cm^–2^, both 1T′-MoS_2_ and 1T′-MoS_2_ are inferior catalysts. Based on the minimum energy consumption required to produce hydrogen at a thermoneutral voltage at 25 °C,^10^ we calculated that the 1T′-MoTe_2_/CC (0.6 mg_1T′-MoTe_2__ cm^–2^) cathode yielded an energy efficiency of 52.2 kW h kg_H2_^–1^ at 150 mA cm^–2^. This is higher than the required <49 kW h kg_H2_^–1^ for 1T′-MoS_2_ (0.4 mg_1T′-MoTe_2__ cm^–2^) based on our estimates at the same current density. An overview of the comparative values is given in Table S7. Although useful for comparison of different types of catalysts, these efficiency values are an oversimplification since they encompass only the factors related to the electrolyzer, while the energy requirements related to running the pumps or keeping the electrolyzer at operational temperature are neglected.

### Electrochemical Stability of 1T′-MoTe_2_/CC in a Single-Cell PEM Water Electrolyzer

The 1T′-MoTe_2_/CC cathode was removed from the electrolyzer and tested by XRD after galvanostatic electrolysis. The peaks associated with the 1T′-monoclinic phase were still present (Figure S21a) and consistent with the XRD pattern of the sample before the electrolyzer testing. This confirmed that the bulk of the sample did not degrade or convert to other polymorphs, consistent with the behavior of related TMDCs in acidic conditions.^46^ However, Raman spectroscopy collected on 1T′-MoTe_2_/CC after galvanostatic electrolysis provided additional information. The band corresponding to the A_g_ mode of 1T′-MoTe_2_ was still observed at ∼160.2 cm^–1^, consistent with the results of XRD. However, two distinct peaks were observed at ∼121.5 and ∼141.8 cm^–1^, attributed to elemental tellurium (Figure 4f), suggesting that a film of Te was formed upon the 1T′-MoTe_2_ film’s surface. The layer of Te must be thin as it was not possible to detect any reflections associated with Te by XRD. The presence of the Te layer was probably the cause for the comparatively low current densities observed in 1T′-MoTe_2_/CC, which explains the drastic difference in performance of 1T′-MoTe_2_ and 1T′-MoS_2_. We attribute the formation of the Te layer in the electrolyzer compared with the half-cell due to the difference in the acidic environment. We hypothesize that the Te layer forms in both single and half-cells; however, in the presence of 1 M H_2_SO_4_, this layer is etched away in the half-cell, making the surface of the electrode fresh. To test this hypothesis, we compared the Te levels determined by ICP-MS analysis in solutions recovered after bulk electrolysis both in the electrolyzer and the three-electrode system (half-cell). While in the single cell, the Te level was only 0.68 ppm, the levels of Te (6 ppm) in the half-cell were 8-fold higher. Thus, in the electrolyzer, the formation of the Te layer prevents leaching, however, at the price of catalytic activity. The previously mentioned postelectrolysis XRD and Raman in the half-cell (Figure S14) show that the 1T′-MoTe_2_ phase is retained after bulk water electrolysis. Therefore, even though in the half-cell ICP confirms catalyst leaching, a freshly formed layer of 1T′-MoTe_2_ acts as the catalyst. The presence of a thin Te layer rather than erosion or degradation of the 1T′-MoTe_2_/CC catalyst was further confirmed by SEM and EDX mapping (Figure S21). The Mo and Te were homogeneously distributed within the film surface without much evidence of delamination.

## Conclusions

In conclusion, it was possible to prepare films of 2D metallic 1T′-MoTe_2_ on a carbon cloth support through a CVD approach, although the growth of phase-pure samples was limited to a maximum loading of 0.6 mg_1T′-MoTe_2__ cm^–2^. When tested in a three-electrode system (using H_2_SO_4_ as an electrolyte), 1T′-MoTe_2_/CC achieved substantially higher current densities at a given overpotential than 2H-MoTe_2_ and mixed-phase 2H/1T′-MoTe_2_ films. Furthermore, in the three-electrode system at 100 mA cm^–2^, the 1T′-MoTe_2_/CC demonstrated only a marginally higher overpotential than leading 1T′-MoS_2_ systems, confirming the literature assessment that the metallic MoTe_2_ appears to be a competent rival to the sulfide as a HER catalyst.

However, when tested as a HER catalyst in a PEM water electrolyzer, 1T′-MoTe_2_ (0.6 mg_1T′-MoTe_2__ cm^–2^) achieved a current density of only 150 mA cm^–2^ at a cell voltage of 2 V. This is far below the values reported on 1T′-MoS_2_ (which achieved >800 mA cm^–2^ at 2 V), suggesting that there are some factors that prevented 1T′-MoTe_2_ from performing. Indeed, the Pt benchmark (0.5 mg_Pt_ cm^–2^) showed substantially better performance (reaching a current density >1000 mA cm^–2^) than 1T′-MoTe_2_. Therefore, the degradation of RuO_2_ OER catalyst was excluded. A possible explanation for the poor catalytic performance of 1T′-MoTe_2_ was provided by the postelectrolysis characterization. The Raman experiments suggested that a layer of Te was formed on the surface of the 1T′-MoTe_2_ catalyst. We hypothesize that this layer passivates the catalytic sites in 1T′-MoTe_2_, ultimately impacting the catalytic activity and preventing the catalyst from operating at its full potential. This pronounced disconnect between the performance of 1T′-MoTe_2_ in a three-electrode configuration versus full electrolyzer testing is important. It confirms that comprehensive, applied validation in an electrolyzer is crucial to the accurate assessment of any emerging PEM electrolysis catalyst.

## Supporting Information Available

Extended description of experimental methods; chronoamperometry profiles; XRD pattern; Raman spectra; SEM/EDX elemental compositions; and electrochemical analysis of 2H; 2H/1T′-MoTe_2_ films; electrolyzer assembly; and postelectrolysis characterizations of films (PDF)

## Abbreviations

TMDCs: two-dimensional transition-metal dichalcogenides
PEM: proton exchange membrane
HER: hydrogen evolution reaction
OER: oxygen evolution reaction
XRD: X-ray diffraction
SEM: scanning electron microscopy
EDXS: energy-dispersive X-ray spectroscopy
LSV: linear sweep voltammetry
