# Supplementary material for: Assessing Challenges of 2D-Molybdenum Ditelluride for Efficient Hydrogen Generation in a Full-Scale Proton Exchange Membrane (PEM) Water Electrolyzer
Source: ACS Sustain Chem Eng. 2024 Jan 6;12(3):1276–85. doi: 10.1021/acssuschemeng.3c06616 (PMC10806992; doi:10.1021/acssuschemeng.3c06616)
Supplement: Supplementary file 1 — sc3c06616_si_001.pdf [file sc3c06616_si_001.pdf]

Supporting Information

Assessing Challenges of 2D-Molybdenum Ditelluride for Efficient Hydrogen Generation in a Full-Scale Proton Exchange Membrane (PEM) Water Electrolyzer

Arun Kumar Samuel,[†] Abdulhai H. Faqeeh,[†,‡] Weihao Li,[†] Zeliha Ertekin,[†] Yuanshen Wang,[†] Jingyi Zhang,[§] Nikolaj Gadegaard,[§] David A. J. Moran,[§] Mark D. Symes[†] and Alexey Y. Ganin[*,†]

[†]School of Chemistry, University of Glasgow, Glasgow G12 8QQ, U.K.

[‡]Department of Chemistry, King Khalid University, Guraiger, Abha 62529, Saudi Arabia

[§]School of Engineering, University of Glasgow, Glasgow G12 8LT, U.K.

[*]Email: alexey.ganin@glasgow.ac.uk.

Number of Pages – S32

Number of Figures – S21

Number of Tables – S7

Supplementary Note 1: Additional Experimental details and Methods

Synthesis of MoO_3 on carbon cloth support

Ammonium molybdate tetrahydrate $(NH_4)_6Mo_7O_{24}\cdot 4H_2O$ (Alfa Aesar, 99%), carbon cloth (0.360 mm thickness) without microporous layer (W0S1011, Fuel Cell Store) and hydrochloric acid (HCl, Sigma-Aldrich, 38%) were used as supplied without further purification. For the preparation of electrolyte, 5 mM of $(NH_4)_6Mo_7O_{24}\cdot 4H_2O$ and 5 ml of HCl were dissolved in 100 ml of deionized water on stirring over 30 mins. The resultant transparent solution was used as an electrolyte for the electrodeposition of MoO_3. Before the electrodeposition process, carbon-cloth was cleaned using acetone (Fisher Scientific, 99%) for 30 mins and then for 5 mins in nitric acid (Fisher Scientific, 70 %).

In the typical electrodeposition experiment, the carbon cloth was used as a working electrode. It was clipped so that the required size area stayed immersed into the electrolyte. Ag/AgCl (3M NaCl saturated, CHI Instruments) and carbon felt (Alfa Aesar, 99%) were used as a reference electrode and as a counter-electrode respectively. The electrodeposition was carried out in Biologic (SP-150) electrochemical workstation at an optimized potential of -0.8 V, vs Ag/AgCl for 300 sec to attain uniform deposition. The electrodeposited substrates were washed with de-ionized water, dried at 60 °C for 30 min and further heated at 450 °C in a muffle box furnace for 3 hours with a heating rate of 5 °C min^{-1}.

Fabrication of the $MoTe_2$ films on carbon cloth by CVD

$MoTe_2$ films on carbon cloth were prepared by conversion reaction of MoO_3 on the carbon cloth. The MoO_3/CC sample and pre-weighted $FeTe_2$ pellet (1.3 cm diameter) used as Te source were placed into 25.5 cm quartz tube (ID: 3.5 cm, OD: 4 cm). The distance between the substrate and the $FeTe_2$ pellet was kept ~ 4 cm. The tube was loaded into in a house-built CVD reactor consisting of a 100 cm long quartz tube (ID: 4 cm, OD: 4.5 cm) placed into a split-zone

high-temperature furnace. The heating rate was maintained at 5 °C min^{-1} with a dwell time of 4 hours, before cooling to room temperature at 5 °C min^{-1}. Carrier gas was a mixture of 5% H_2 in Ar (BOC) at a flow rate of ca. 125 sccm.

Iron-telluride ($FeTe_2$) powder was used as a tellurium (Te) source for the preparation of $MoTe_2$. $FeTe_2$ powder was synthesized by a typical solid-state reaction method. The stoichiometric ratio of iron (metal basis, Alfa Aesar, ~99.9%) and tellurium (metal basis, Alfa Aesar, ~99.9%) powders were taken and grounded inside the Ar-filled glove box (MBraun, <0.1 ppm O_2) for 15 mins with pestle and mortar. The resultant powder was transferred into an alumina boat and heated at 500 °C for 14 hrs under 50 sccm in a mixture of H_2/Ar (H_2 5%, Ar 95%, BOC) gas flow at a heating rate of 3 ºC min^{-1} and cooled to room temperature at 5 ºC min^{-1}.

Electrochemical measurements in a three-electrode configuration

All the electrochemical experiments were performed using a Biologic SP-150 potentiostat (EC Labs) in a three-electrode configuration: $MoTe_2$/CC substrate as a working electrode, 3M Ag/AgCl as a reference electrode and carbon-felt as a counter electrode. Aqueous 1M H_2SO_4 was used as an electrolyte. Linear sweep voltammetry (LSV) polarization measurements were performed within 0.1 V to –0.8 V range at a scan rate of 5 mV s^{-1}, under constant stirring of 350 rpm. Nitrogen gas (BOC, 99.9%) was purged during the experiment. Tafel plots were obtained from LSV polarization with constant stirring at scan rates of 5 mV s^{-1}. Cyclic voltammetry measurements (CV) were performed from 0.05 V to –0.3 V (vs RHE) at different scan rates to measure the double layer capacitance. The electrochemical active surface area (ECSA) was determined by performing potential sweeps within a narrow potential range in the non-Faradaic region. Chronoamperometry (CA) was performed using an identical three-electrode set up at a constant potential depending on the current densities required to achieve.

Electrochemical impedance (EIS) experiments were performed at a selected cathodic overpotential for all the electrodes in the frequency range of 200 kHz to 1 mHz. All the experimental electrode potentials were converted to RHE scale as follows. $E_{RHE} = E^{\circ}_{Ag/AgCl} + E_{Ag/AgCl} + 0.059$ pH, where $E_{Ag/AgCl}$ is experimental potential, $E^{\circ}_{Ag/AgCl}$ is 0.209 V (3M Ag/AgCl reference electrode), and the pH is ~1. The compensation for the ohmic resistance involved subtracting 85% of the voltage drop it induced. The experimental errors were determined through a minimum of three independent measurements, and the discrepancies between these measurements were considered as errors.

Fabrication of RuO_2 / TiO_2 microporous layer anodes

A titanium felt (porosity, 53−56%, thickness 0.25 ± 0.05 mm, Fuel Cell Store) was employed as the anode gas diffusion layer. The felt (2.3×2.3 cm^2) was sprayed with Ti-particles to form a microporous layer (MPL).[1] The MPL suspension was made by weighing 2.5 g of Ti-particles (5 μm, US Nanomaterials Research) into a vial placed in a N_2-regulated glove box, preventing any possible combustion or oxidation of Ti microparticles. The vial was then sealed with a septum and removed from the glove box. Subsequently, 2.5 mg of Nafion (5 wt. %, Sigma Aldrich) and 10 g of isopropanol were added to the vial. Then, the suspension was sonicated for 15 min, followed by an additional 1 hour sonication after the addition of 10 g of ethylene glycol. The Ti microporous layer suspension was sprayed onto the Ti-fiber felt (loading mass of ~1.8 mg cm^{-2}) using an AB-182 double action suction-feed airbrush (0.5 mm nozzle, Everything Airbrush, UK).

Subsequently, the Ti felt coated with the Ti-microporous layer was sprayed with the RuO_2 ink with the loading mass of ~1.8 mg cm^{-2} using another AB-182 double action suction-feed airbrush. The ink was prepared by mixing Nafion solution (5 wt.%, Sigma-Aldrich), RuO_2 powder (99% anhydrous, Thermo Scientific) and carbon black (99.9+%, Thermo Scientific) in

15: 20 : 65 mass ratio respectively. After mixing the ink components using isopropyl alcohol, the ink was sonicated for 2.5 hours in the ultrasonication bath (ice bath was used to prevent the evaporation of the isopropyl alcohol). Finally, the spray coated anode gas diffusion electrode was placed into a preheated to 120 °C oven and dwelled for 1 h. After that it was taken to ambient.

A single-cell PEM water electrolyzer components

Two individual 5 cm^2 Ti-flow fields with serpentine channel (T3, v2.0, Dioxide Materials) were used as anode and cathode plates. On the cathode side, metallic 1T'-$MoTe_2$ film on the CC (2.3×2.3 cm^2) was used as a catalyst and a cathode gas-diffusion electrode. The OER catalyst (RuO_2/carbon/Ti-fiber felt) loaded gas-diffusion electrode with an active area of about 5.3 cm^2 (2.3×2.3 cm^2) was used as an anode. The anode and cathode gaskets (PTFE, Fuel Cell Store) of two-different thickness of 0.005"and 0.010" with the size of 2.5×2.5 cm^2 were used for the assembly. The pre-treated Nafion (N-117, Fuel Cell Stores) was used as a proton-exchange membrane (PEM) inside the assembly. The membrane pre-treatment was carried out separately by the following procedure. Initially, 2.4×2.4 cm^2 of Nafion membrane was placed inside the pre-heated 1M H_2SO_4 (Fisher Scientific, ~95%) solution at 80 ºC for 1 hour. The treated membrane was transferred again to pre-heated DI water bath at 80 ºC for 1 hour and stored in a container containing DI water.

Electrolyzer Assembly

Initially, 0.005" thick PTFE gasket was assembled on the top of the Ti-flow channel anode plate. The OER gas diffusion electrode was placed on the anode Ti-flow field serpentine channel, without any overlay on the anode gasket. The pre-treated Nafion membrane was used as a separator between the anode and cathode gas diffusion layers inside the assembly. After

placing the Nafion membrane on the top of the OER-GDL (RuO_2/Ti-fiber felt), the cathode-GDL (1T'-$MoTe_2$/CC) was placed followed by cathode PTFE gasket without any miss alignment. The rubber O-ring was placed into the groove of both the anode and cathode Ti-flow field channels with flow facing down on top of the assembly. The flat washers and nuts (Dioxide Materials) were inserted and tightened diagonally up to 35 in-lb force using torque wrench and Allen key. After the OER and HER electrodes assembly, the electrolyzer was taken for leak-proof testing for 30 mins and further subjected for full-scale testing at 60 °C at a flow rate of 18 mL min^{-1}. The temperature of both anodic (T_1) and cathodic (T_2) water reservoirs were monitored using TC-08 thermocouple data logger (Pico Technology).

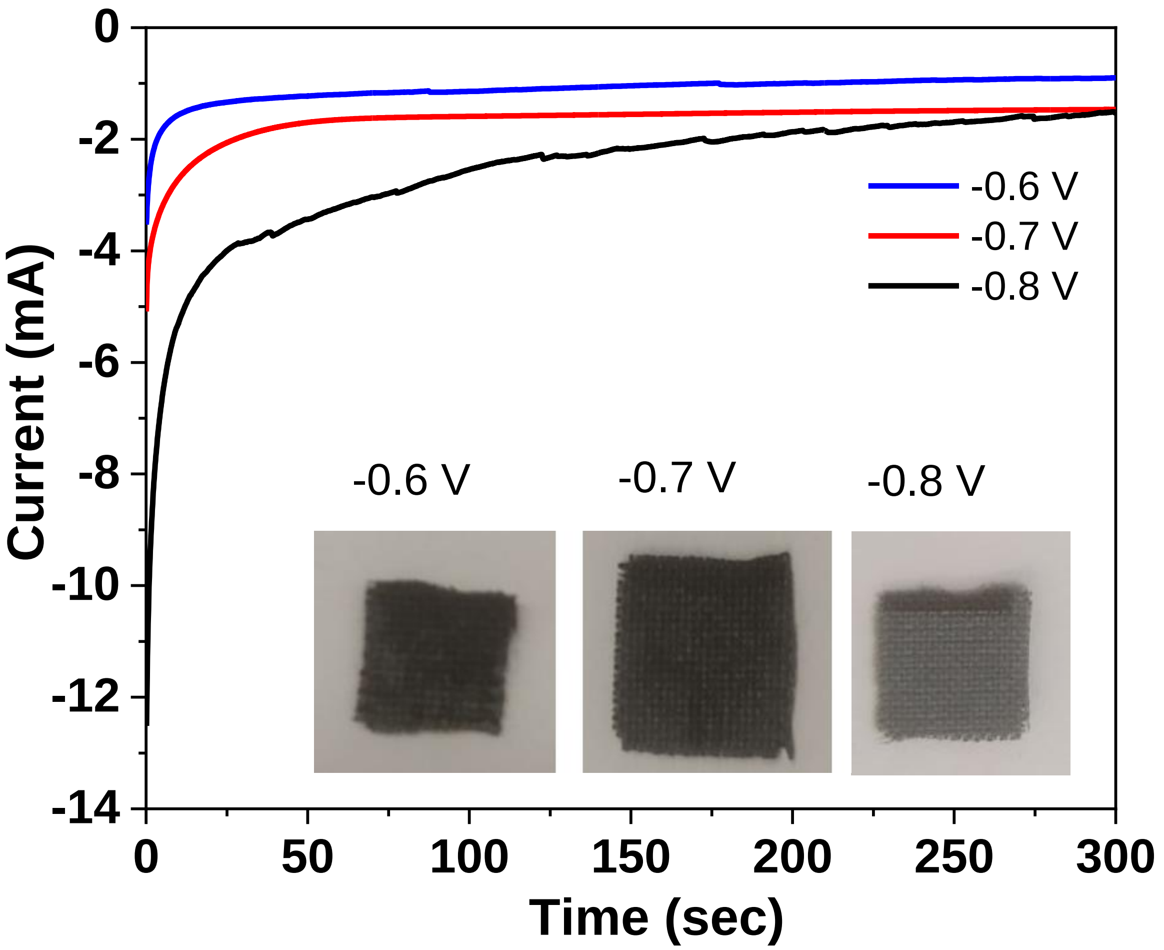

Figure S1. Chronoamperometry profiles of the electrodeposited MoO_3 on carbon cloth at different cathodic potentials. The inset shows optical images of the produced films. The experiment performed at -0.8 V for 300 sec resulted in homogeneous MoO_3 films with good surface coverage.

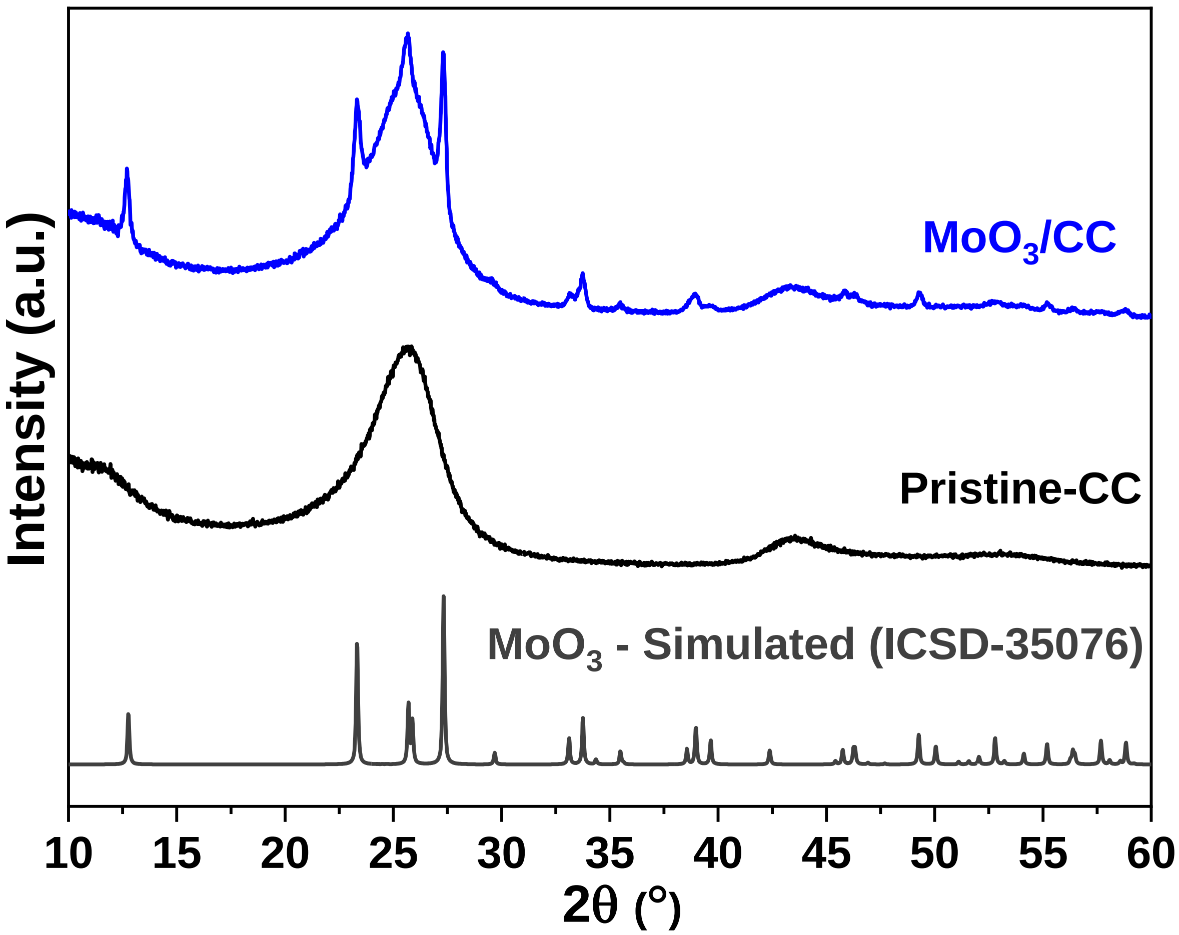

Figure S2. XRD patterns of the pristine carbon cloth (CC) and the electrodeposited MoO_3 / CC in comparison with the simulated pattern based on ICSD data.

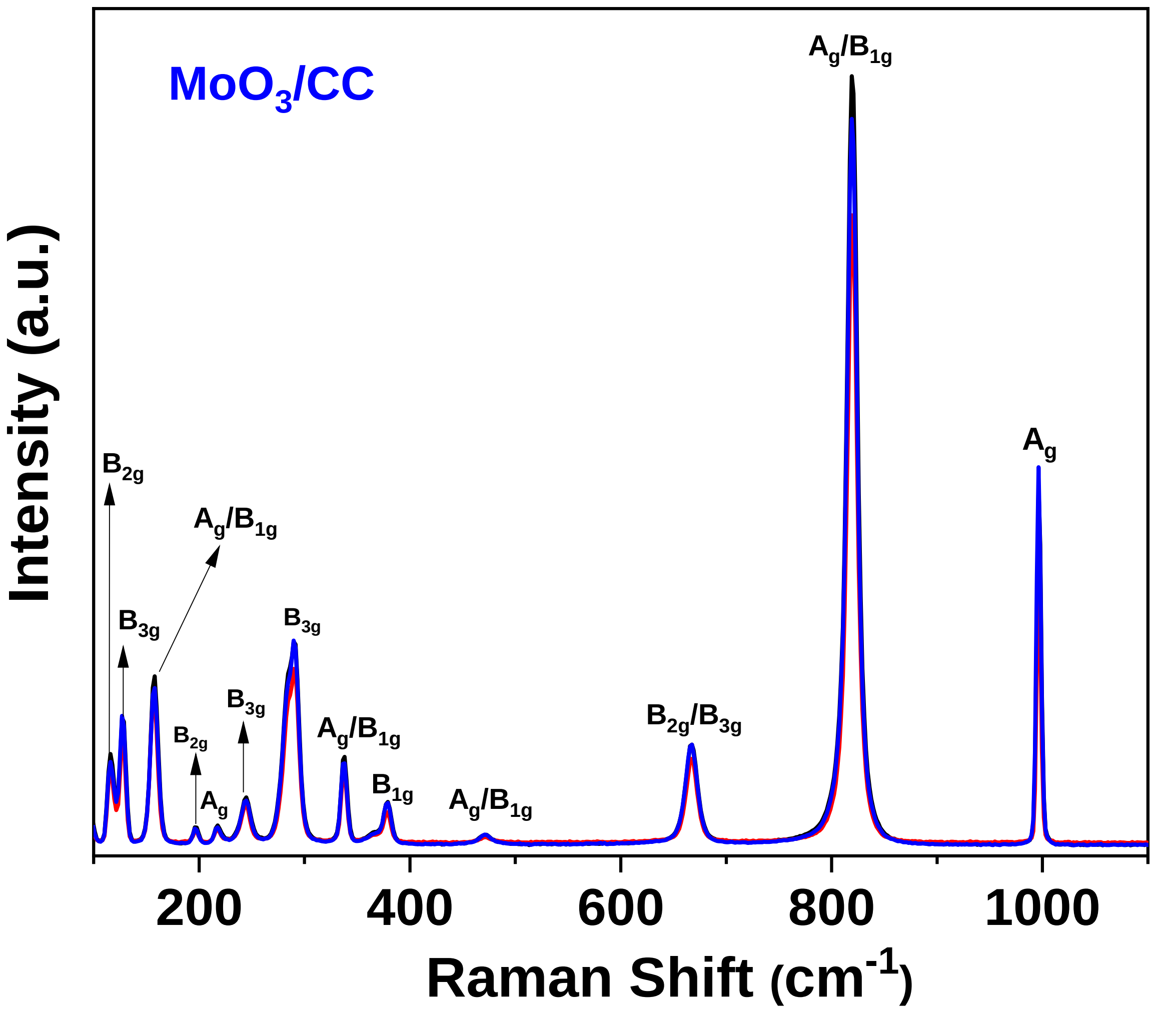

Figure S3. Raman spectra of the electrodeposited MoO_3 / CC. Three spectra recorded at three different spots across the MoO_3/CC show nearly perfect overlap confirming the homogenous nature of the MoO_3 films across the carbon cloth substrate.

Table S1. Raman active modes of electrodeposited α-MoO_3 / CC.

**Raman frequency (cm^{-1})**	**Raman modes**	**Peak attribution**
116.3	B_{2g}	Translational chain mode along c-direction (T_c)
127.5	B_{3g}	Translational chain mode along c-direction (T_c)
158.5	A_g/B_{1g}	Translational chain mode along b-direction (T_b)
196.6	B_{2g}	O=Mo=O, twisting mode (τ)
217.6	A_g	Rotational chain mode along c-direction (R_c)
245.6	B_{3g}	O=Mo=O, twisting mode (τ)
290.8	B_{3g}	O=Mo=O, wagging mode (ω)
337.8	A_g/B_{1g}	O−Mo−O, bending mode (δ)
378.5	B_{1g}	Bending mode (δ) with O=Mo=O (scissoring)
471.1	A_g/B_{1g}	O−Mo−O, stretching (ν) and bending (δ) modes
666.5	B_{2g}/B_{3g}	O−Mo−O, stretching (ν) mode
819.2	A_g/B_{1g}	O=Mo=O, symmetric stretching (ν_s) mode
994.8	A_g	O=Mo=O, asymmetric stretching (ν_{as}) mode

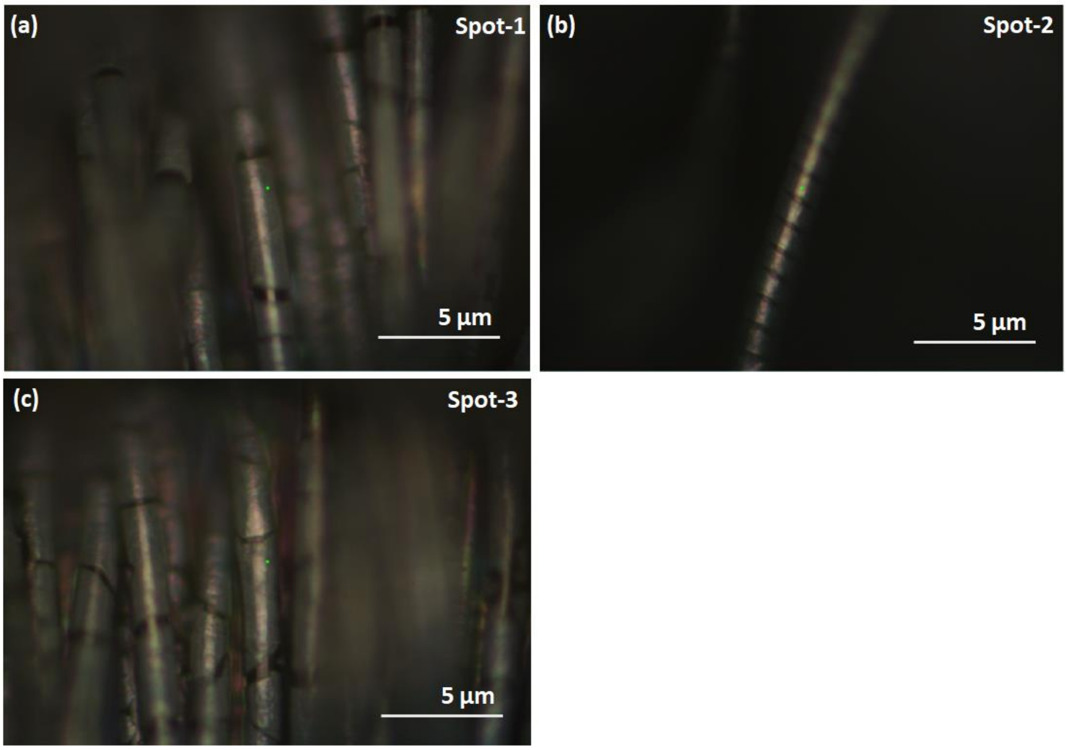

Figure S4. Optical images of MoO_3 film electrodeposited on CC support recorded at three different spots **(a)** spot-1, **(b)** spot-2 and **(c)** spot-3 using 50× objective lens.

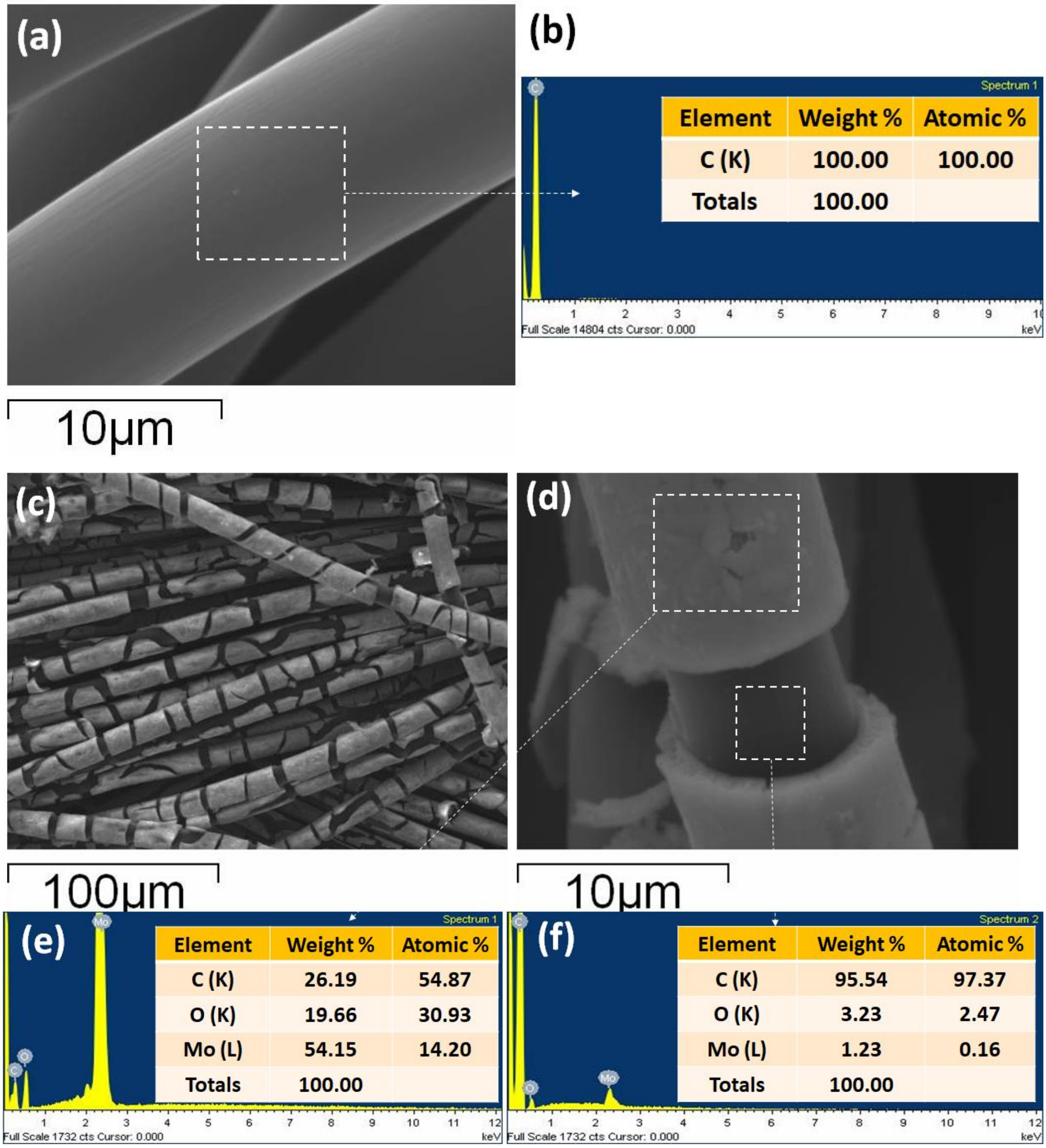

Figure S5. (a) Representative SEM images of pristine CC and **(b)** the EDX spectra of pristine CC. **(c, d)** SEM image of a typical MoO_3 film on CC consisting of two regions. **(e-f)** The EDX spectra showing Mo peak in the film region and predominantly carbon in the thinner region respectively.

Table S2. Experimental conditions of the CVD process used for the growth of $MoTe_2$/CC.

Structural polymorphs	Te Source Temperature (°C)	Substrate Temperature (°C)
2H-$MoTe_2$ /MoO_2	650	665
2H-$MoTe_2$/CC	700	718
2H/1T'-$MoTe_2$/CC	730	750
2H/1T'-$MoTe_2$/CC	750	775
1T'/2H-$MoTe_2$/CC	780	800
1T'-$MoTe_2$/CC	800	830

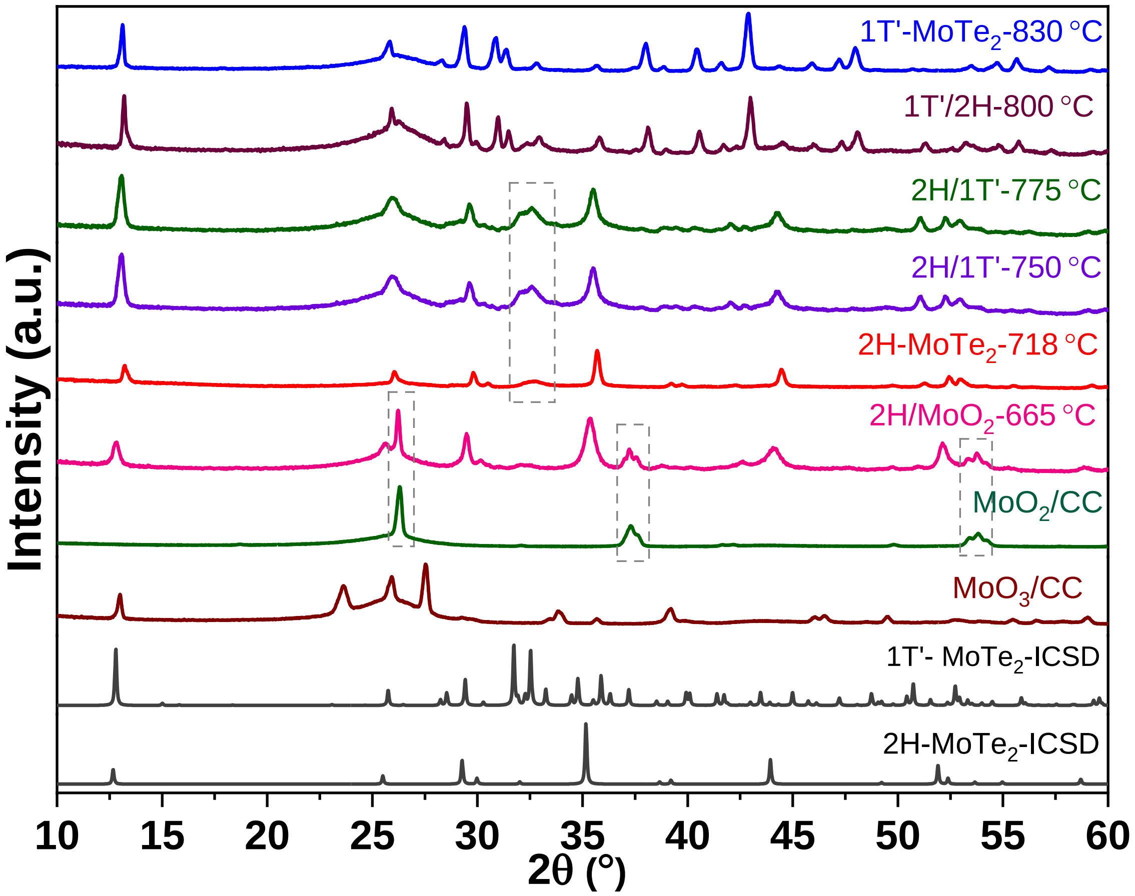

Figure S6. XRD patterns of $MoTe_2$ films formed on carbon cloth depending on the reaction temperatures within the CVD setup. Experimental XRD patterns were compared with the simulated based on standard ICSD data of 1T'-$MoTe_2$ and 2H-$MoTe_2$. Films prepared at 665 °C contained 2H-$MoTe_2$ and monoclinic MoO_2 phases. The presence of the MoO_2 phase suggested incomplete conversion to $MoTe_2$. Pure 2H-$MoTe_2$ forms at 718 °C, while between 750 - 800 °C mixed phase samples consisting of 1T'-$MoTe_2$ and 2H-polymorphs in different ratio were formed.

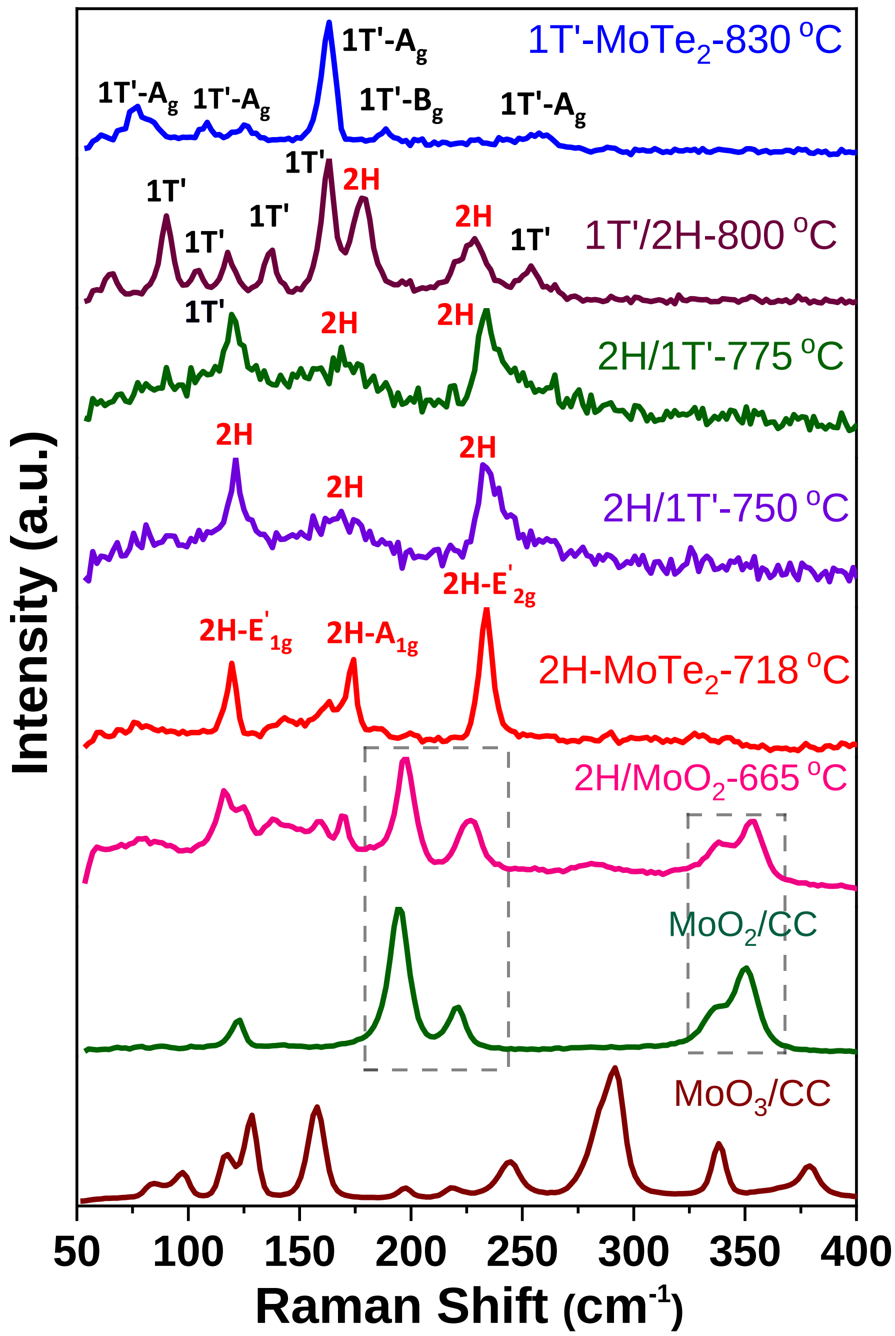

Figure S7. Raman spectra collected on $MoTe_2$ films formed on carbon cloth by CVD at different temperatures. Raman peaks were compared with MoO_3 and MoO_2 grown on CC support as well as peaks for 2H- and 1T'-$MoTe_2$ phases. Pure 2H-$MoTe_2$ formed at 718 °C, while between 750 - 800 °C mixed phase samples consisting of 1T'- and 2H $MoTe_2$ polymorphs in different ratio were formed.

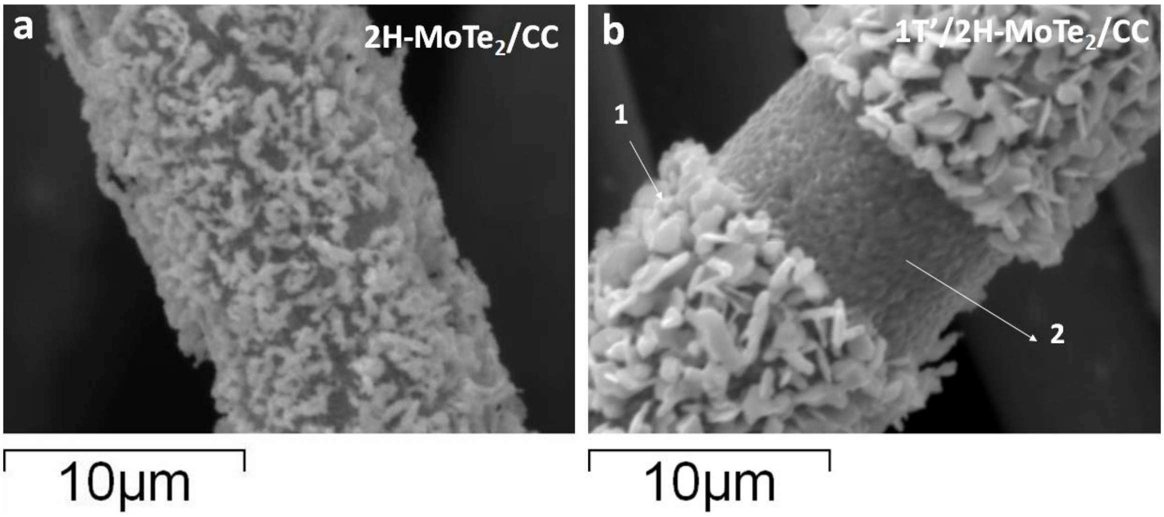

Figure S8. SEM images of **(a)** 2H-$MoTe_2$ on CC displaying small crystallites aggregated over the CC surface with a greater number of edge sites exposed. The relevant EDX experiment an average composition $MoTe_{2.19\pm0.44}$. **(b)** Mixed 1T'/2H'-$MoTe_2$ films. In line with two-phased nature of 1T'/2H-$MoTe_2$/CC film, display two different morphologies in the SEM images. In the region 1, the particles are aggregated in the form of platelets, whilst the region 2 shows a homogenous film.

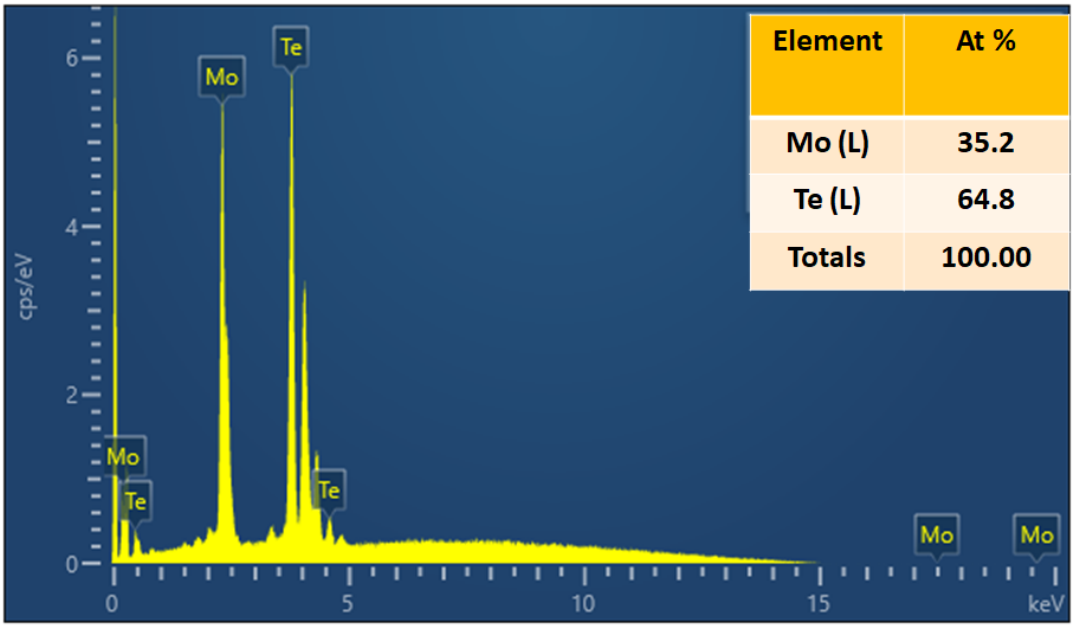

Figure S9. A typical EDX spectrum collected on the 1T'-$MoTe_2$ / CC films prepared at 830 °C. The insert gives a Mo / Te atomic ratio.

Table S3. EDX Elemental analysis of $MoTe_2$ /CC films. Standard deviations were calculated based on averaging the EDX results on at least five points collected at different region of the CC substrate.

	**Temp (ºC)**	**Mo, at. %**	**Te, at. %**	**Te/Mo**
Theoretical	-	33.33	66.67	2
2H-$MoTe_2$/CC	718	31.82±4.26	68.26±4.18	2.19±0.44
1T'/2H-$MoTe_2$/CC	800	36.84±5.5	62.56±5	1.80±0.3
1T'-$MoTe_2$/CC	830	34.73±1.82	64.67±1.40	1.93±0.10

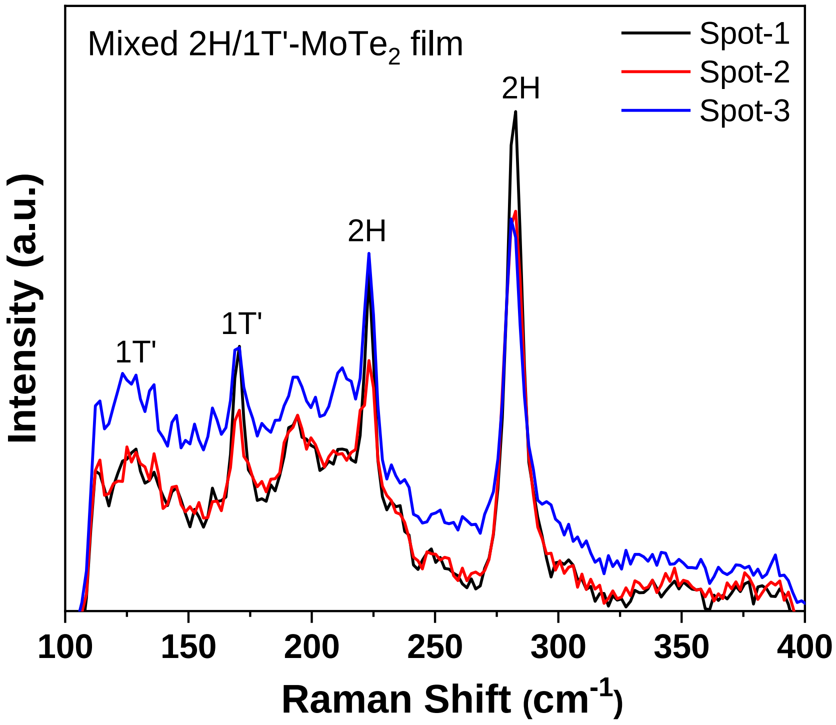

Figure S10. The Raman spectra collected on films formed on carbon cloth at 850 ºC corresponding to loading of 1.8 mg cm^{-2} revealing mixed 1T' and 2H-$MoTe_2$ samples instead of pure 1T'-$MoTe_2$.

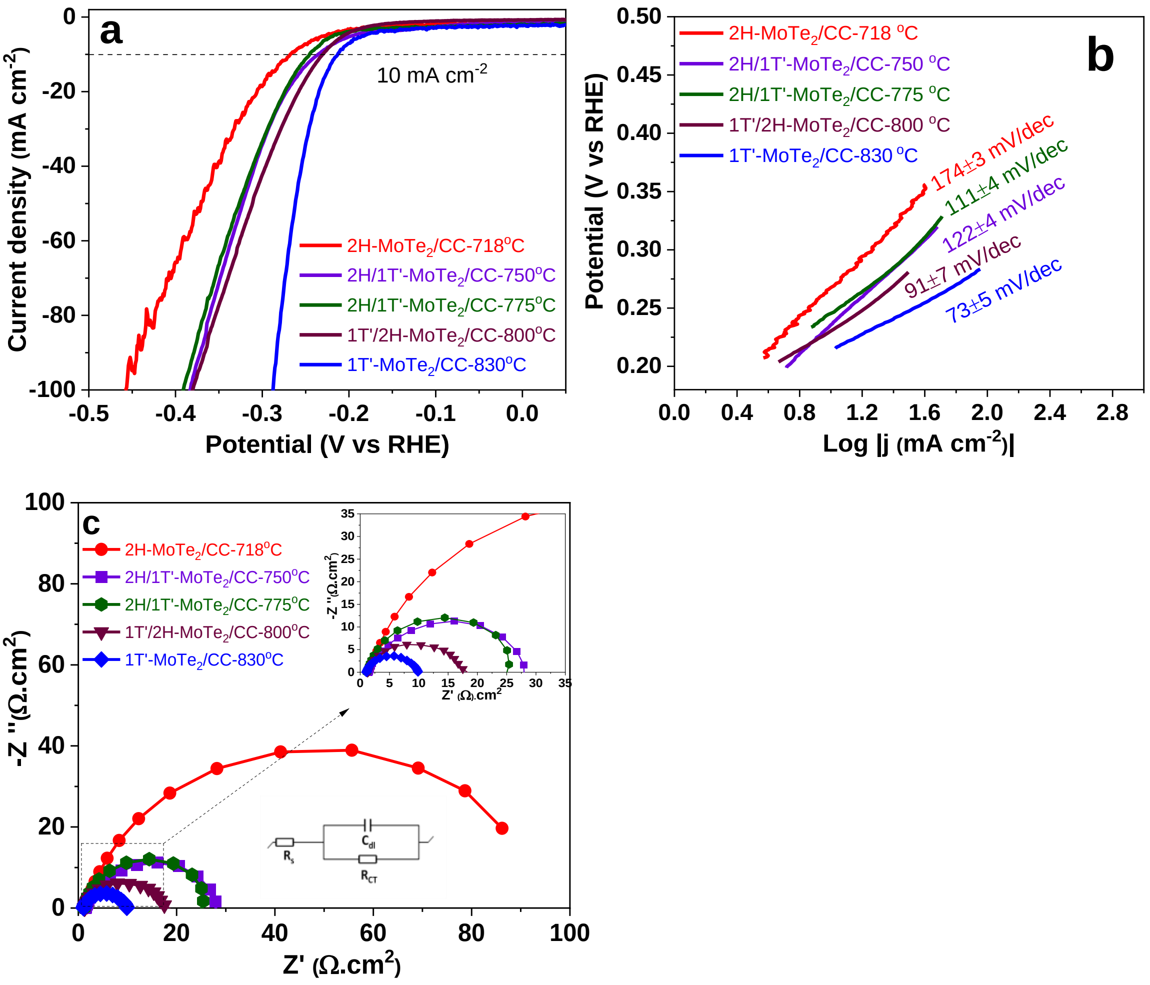

Figure S11. The zoomed in areas and extended data related to electrochemical performance of $MoTe_2$/CC films. **(a)** LSV polarization curve. **(b)** Tafel plots and equivalent Tafel slopes values extracted from LSV polarization data at different overpotential range of $MoTe_2$ polymorphs immobilised on CC support at different annealing temperatures. **(c)** Nyquist plot of $MoTe_2$ polymorphs measured at different annealing temperatures.

Table S4. Overpotential values of $MoTe_2$ / CC at different current densities.

**Structural polymorphs**	**Reaction temp (^oC)**	**η, mV (10 mA cm^{-2})**	**η, mV (50 mA cm^{-2})**	**η, mV (100 mA cm^{-2})**	**η, mV (200 mA cm^{-2})**
2H-$MoTe_2$/CC	718	-283 ± 13	-378 ± 6	-457 ± 7	-
2H/1T'-$MoTe_2$	750	-250 ± 3	-331 ± 5	-386 ± 8	-506 ± 7
2H/1T'-$MoTe_2$	775	-255 ± 2	-327 ± 6	-407 ± 4	-521 ± 2
1T'/2H-$MoTe_2$	800	-244± 3	-314 ± 5	-394 ± 7	-497 ± 5
1T'-$MoTe_2$	830	-212 ± 3	-261± 2	-290 ± 4	-325 ± 3

Table S5. Comparison of HER electrochemical catalytic performance of $MoTe_2$ films on carbon cloth in a 3-electrode system with the literature.

**Catalyst**	**Active area**	**Preparation method**	**Electrolyte**	**η (mV) at 10 mA cm^{-2}**	**Tafel slope (mV dec^{-1})**	**Ref**
2H-$MoTe_2$	1×1 cm^2	Electrodeposition & CVD	1M H_2SO_4	-283 ± 13	174 ± 3	This work
1T'/2H-$MoTe_2$	1×1 cm^2	Electrodeposition & CVD	1M H_2SO_4	-255 ± 2	91 ± 7	This work
1T'-$MoTe_2$	1×1 cm^2	Electrodeposition & CVD	1M H_2SO_4	-212 ± 3	73 ± 5	This work
1T'-$MoTe_2$	0.4 cm^2	CVD	1M H_2SO_4	-230	127	Ref. [2]

Table S6. Electrochemical properties of $MoTe_2$ / CC films. A literature value of specific capacitance of 0.035 mF cm^{-2} [Ref. 3] was utilized for ECSA.

**Structural polymorphs**	**Reaction temp (°C)**	**Tafel slope (mV dec^{-1})**	**EIS ($\Omega.cm^2$)**	**C_{dl} (mF cm^{-2})**	**ESCA (cm^2)**
2H-$MoTe_2$/CC	718	174 ± 3	98.53	2.52	71
2H/1T'-$MoTe_2$/CC	750	122 ± 4	26.85	3.34	99
2H/1T'-$MoTe_2$/CC	775	111 ± 4	24.57	4.70	137
1T'/2H-$MoTe_2$/CC	800	91 ± 7	16.65	4.96	150
1T'-$MoTe_2$/CC	830	73 ± 5	8.75	6.73	196

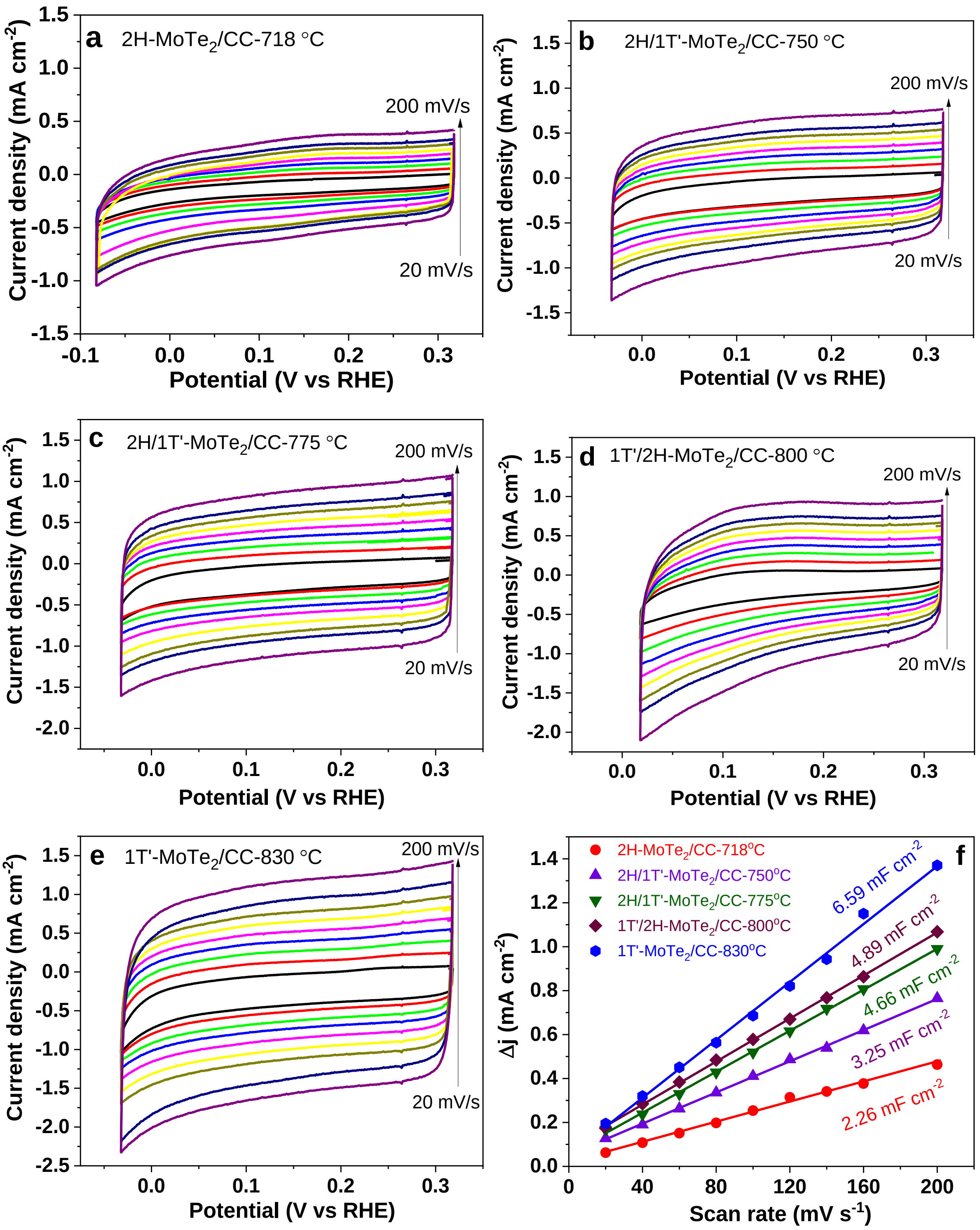

Figure S12. (a-e) Double-layer capacitance (C_{dl}) of $MoTe_2$ / CC films, measured at different scan rates (20, 40, 60, 80, 100, 120, 140, 160, and 200 mV/s). **(f)** Double layer capacitance calculated from current density differences of $MoTe_2$ / CC films plotted against different scan rates (capacitance current difference were measured at 0.15 V (vs. RHE).

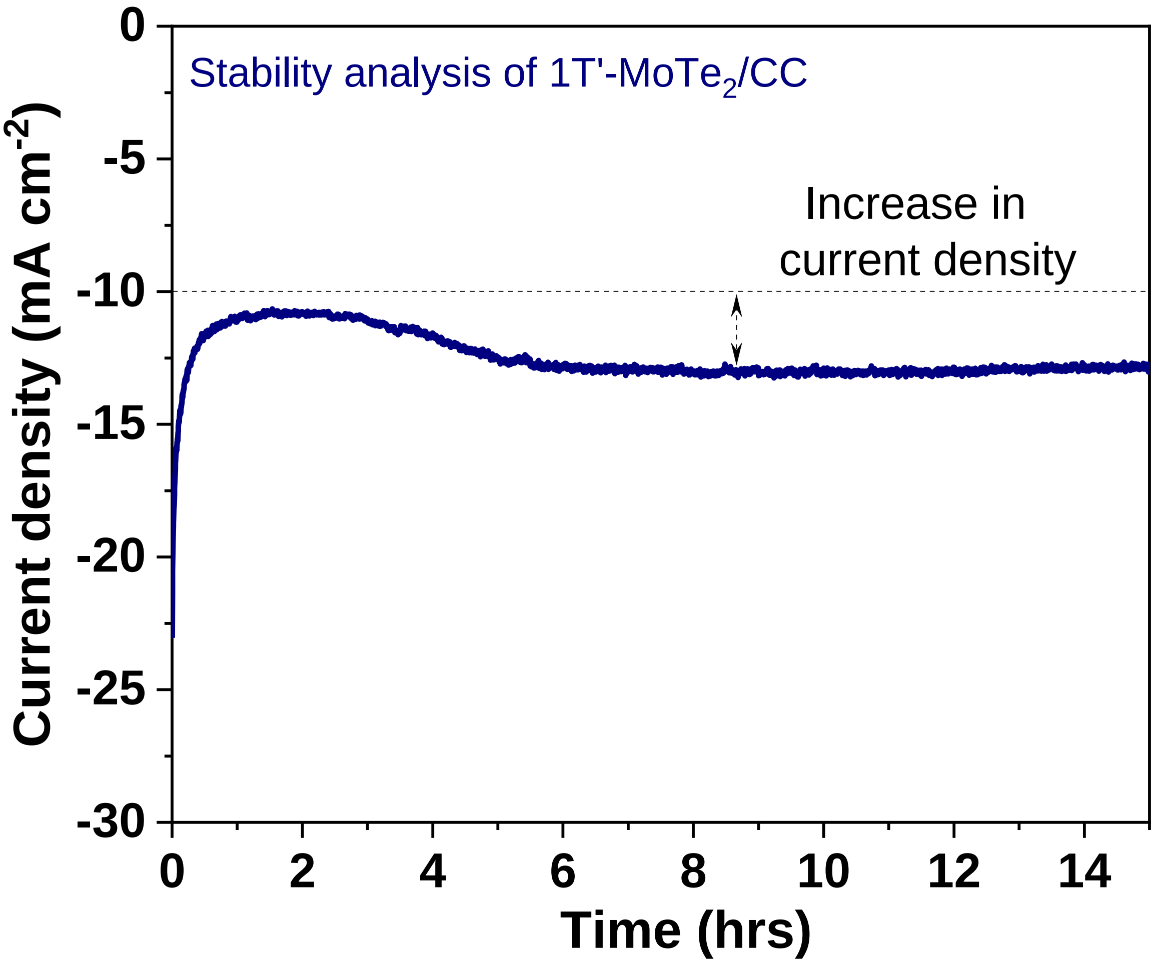

Figure S13. Chronoamperometry profile of 1T'-$MoTe_2$/CC performed in 1M H_2SO_4 aqueous electrolyte at a constant cathode potential of -0.22 V (vs RHE) for 15 hours.

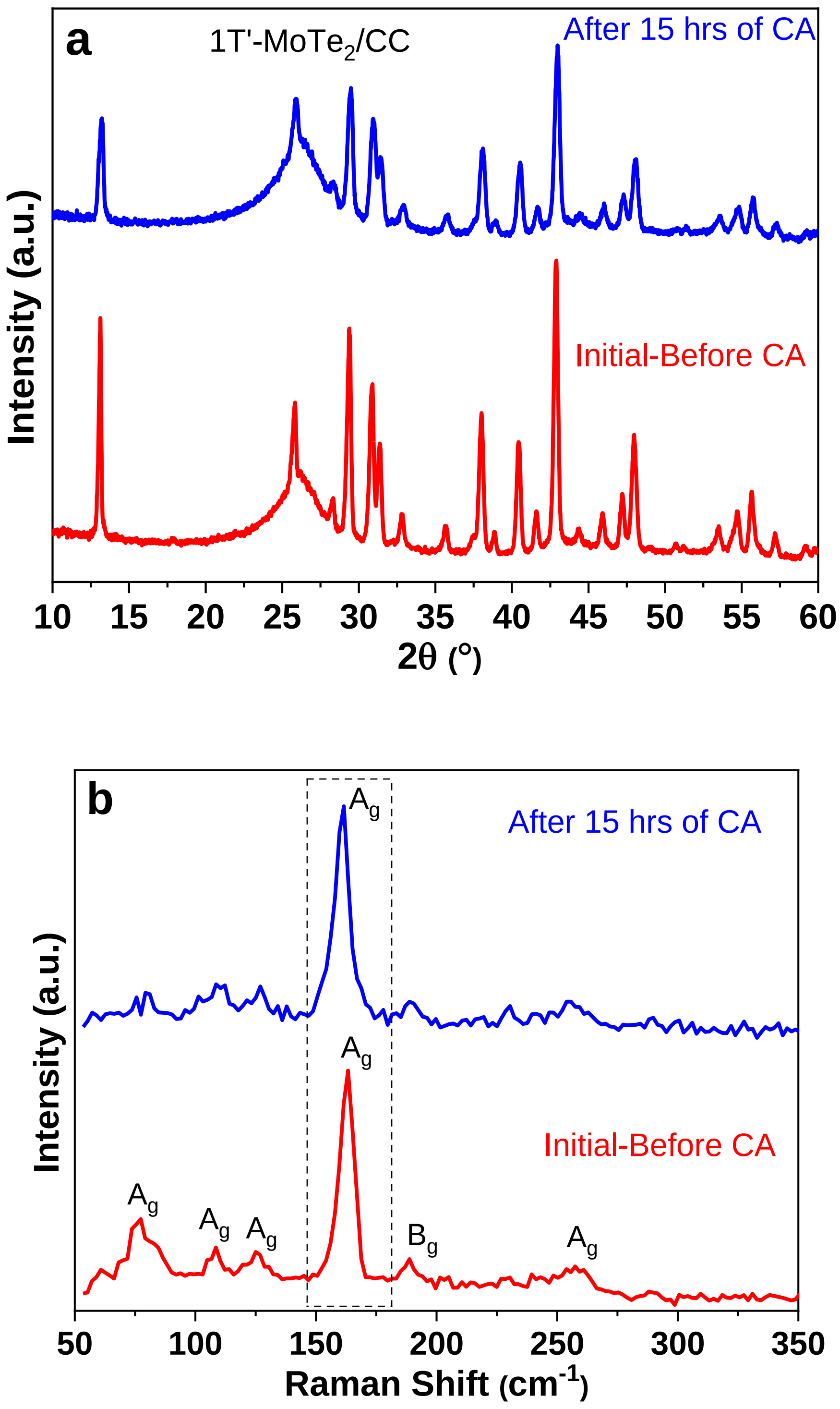

Figure S14. (a) XRD and **(b)** Raman studies performed before and after 15 hours of CP stability test.

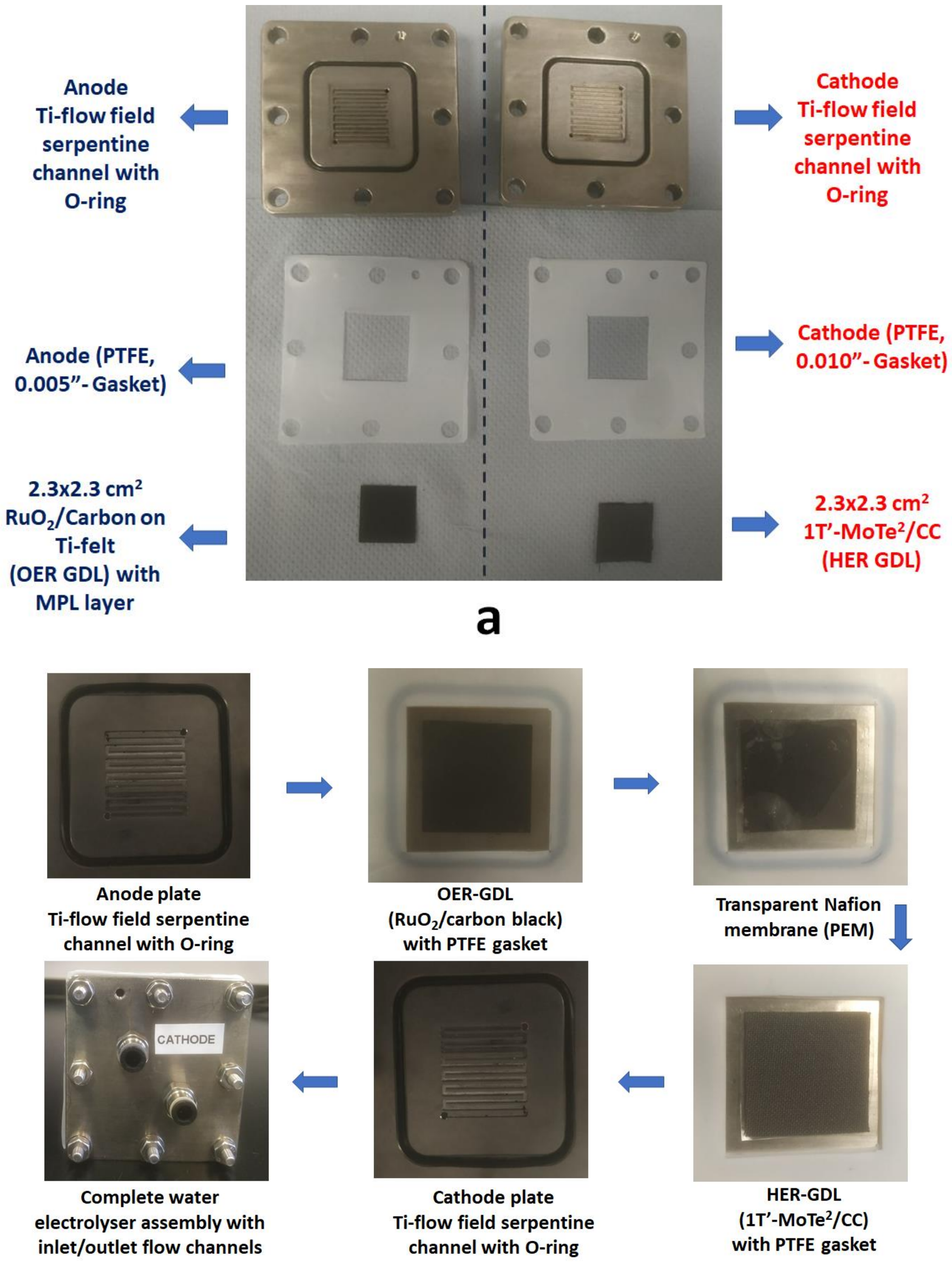

Figure S15. A single-cell PEM flow-cell water electrolyzer assembly. (a) Exploded view and (b) stepwise assembly of the prototype with respective anode and cathode gas-diffusion layers.

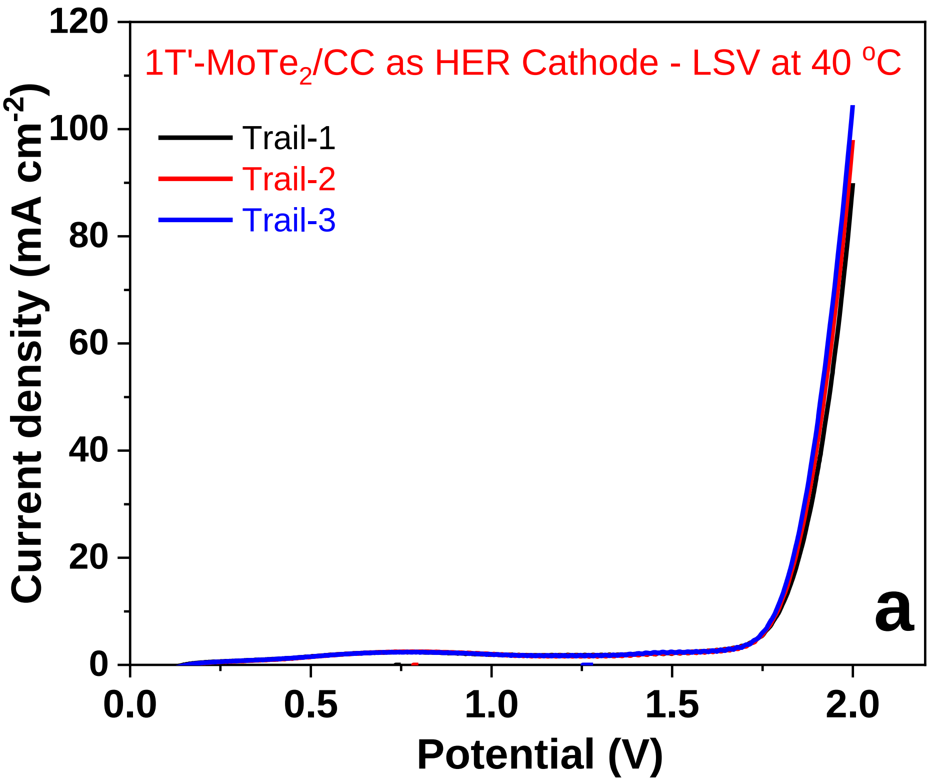

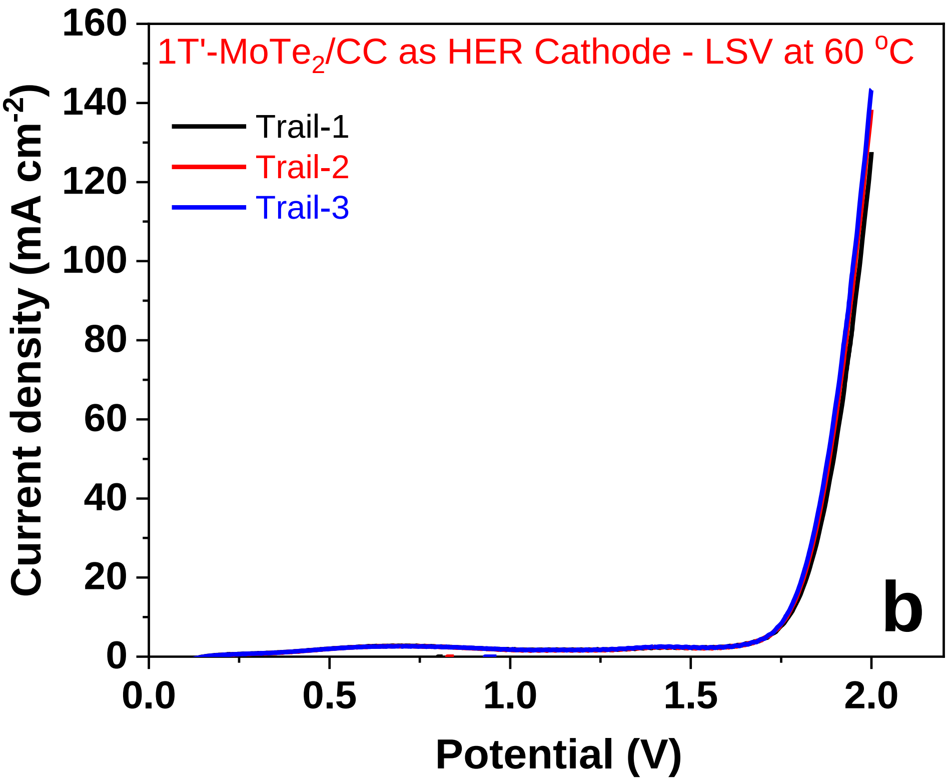

Figure S16. LSV polarization curves of a single-cell PEM flow-cell electrolyzer operated at **(a)** 40 °C and **(b)** 60 °C using 1T'-$MoTe_2$/CC as cathode and RuO_2/Ti-fiber felt as anode.

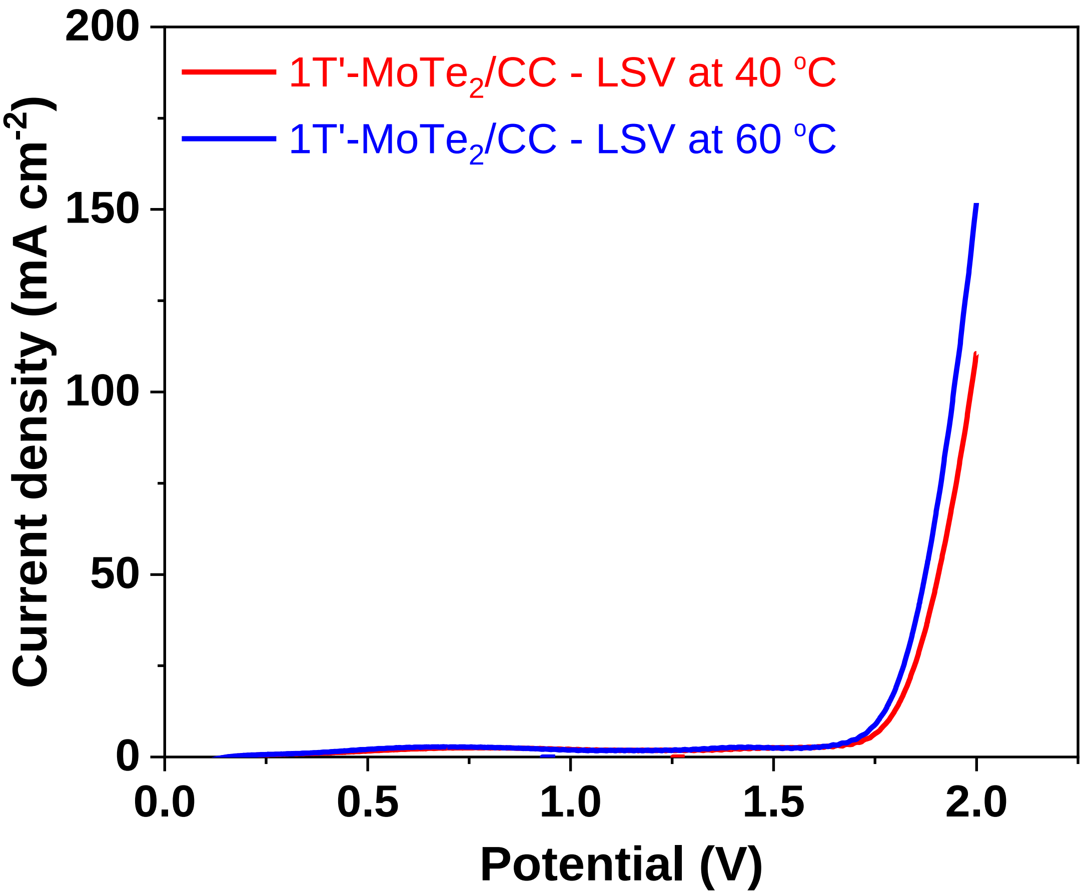

Figure S17. Comparison of LSV polarization curve of a single-cell PEM flow-cell electrolyzer operated at 40 °C and 60 °C using 1T'-$MoTe_2$/CC as cathode and RuO_2/Ti-fiber felt as anode.

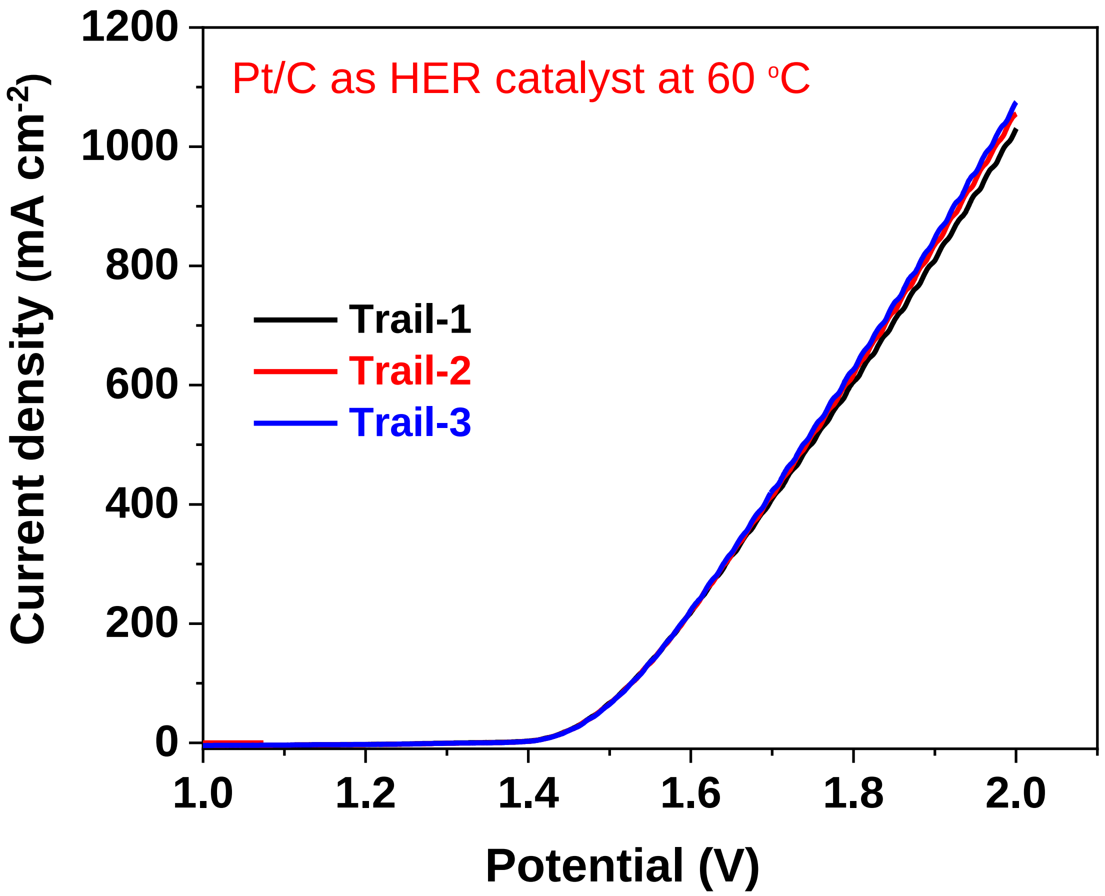

Figure S18. LSV polarization curve of a single-cell PEM flow-cell electrolyzer operated at 60 °C using Pt/C on CC as cathode and RuO_2/Ti-fiber felt as anode.

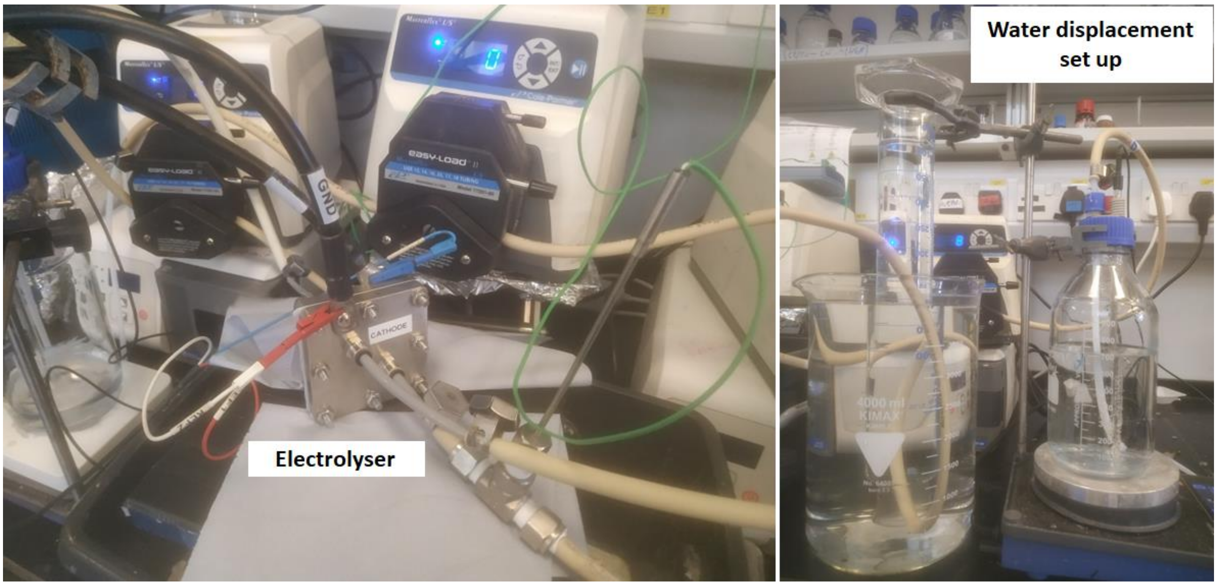

Figure S19. A single-cell 5 cm^2 PEM flow-cell water electrolyzer connected to water-gas displacement set up. Faradaic efficiency was calculated from charge passed, and the proportion of H_2 measured experimentally was determined using the water-gas displacement method.

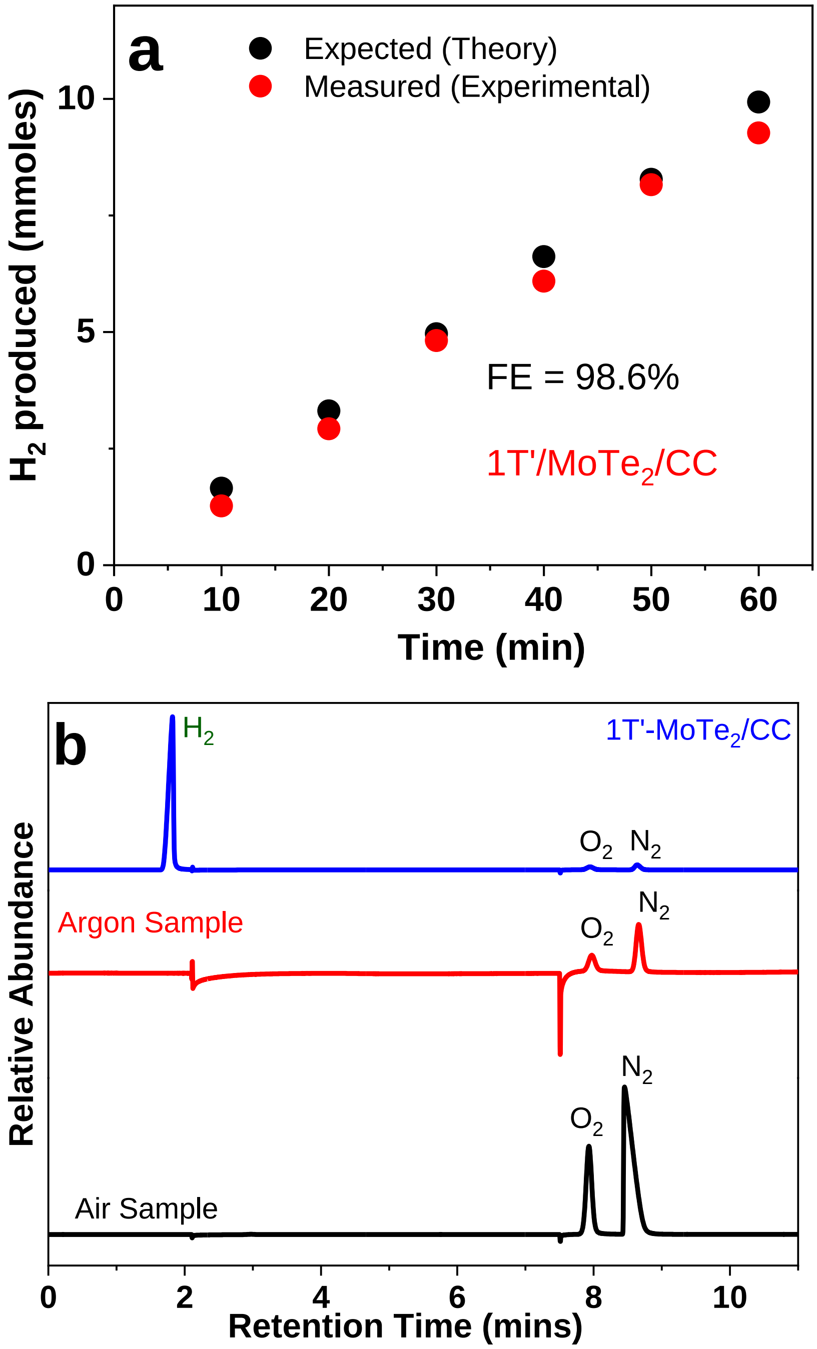

Figure S20. (a) Faradaic efficiency estimated from the water-gas displacement after galvanostatic electrolysis at a constant applied current density of 100 mA cm^{-2} for 60 min. **(b)** Gas chromatography was conducted to verify the presence of H_2 gas collected from the 5 cm^2 PEM flow-cell water electrolyzer using 1T'-$MoTe_2$ film as a cathode. Prior to the experiment, the gas chromatograph was calibrated using air samples and Ar-gas to eliminate effects of air leaks.

Table S7. A summary of 5 cm^2 PEM electrolyzer: Cell specifications and operational parameters comparison 1T'-MoS_2 electrocatalyst as cathodes in PEM electrolyzer.

**PEM Electrolyzer**	**This work**	[4]	[5]
Cell size	5 cm^2 – single cell	5 cm^2 – single cell	5 cm^2 – single cell
OER GDL	RuO_2/Carbon on Ti-fibre felt electrode	RuO_2/Carbon on Ti-fibre felt electrode	$IrRuO_x$ on Ti electrode
HER GDL	1T'-$MoTe_2$ on carbon cloth	1T'-MoS_2 powder on carbon fiber paper	1T'-MoS_2 powder on carbon fiber paper
OER / HER – GDL active area	5.29 cm^2	5 cm^2	5 cm^2
Membrane	Nafion-117 (commercial)	Nafion-117 (commercial)	Nafion-115 (commercial)
Cell flow-rate	18 ml mim^{-1}	N/A	20 ml mim^{-1}
Cell operation temperature	60 °C	80 °C	60 °C
Cell voltage	1.96 V	1.94 V	1.96 V
Catalyst loading	0.6 mg/cm^2	0.8 mg/cm^2	0.14 mg/cm^2
Current density at 2V from LSV	~0.15 A cm^{-2}	~1 A cm^{-2}	~0.8 A cm^{-2}

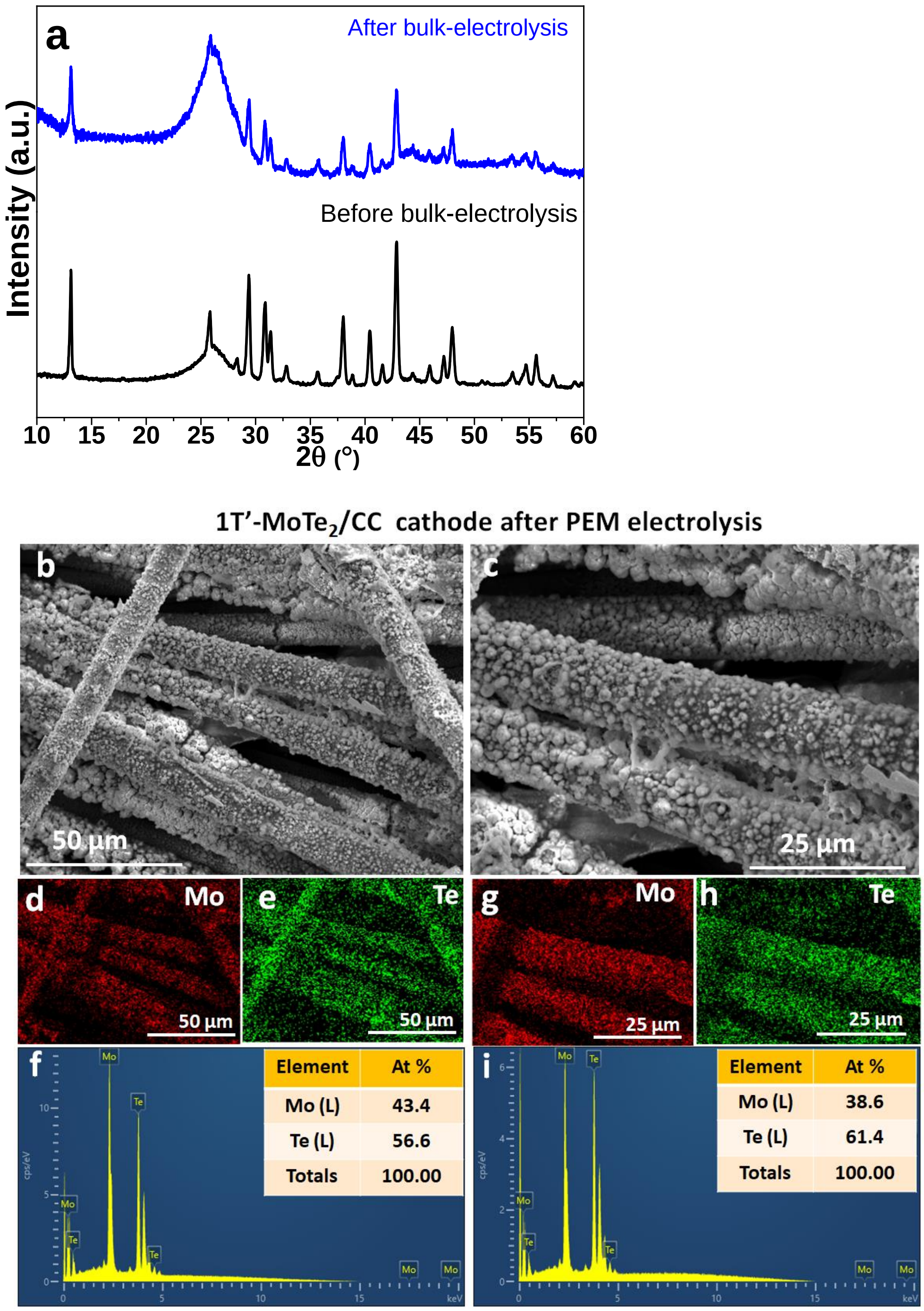

Figure S21. (a) XRD pattern of 1T'-$MoTe_2$/CC catalyst before and after tests in a PEM electrolyzer operated at 60 °C. **(b-c)** Low and high magnification SEM image of 1T'-$MoTe_2$/CC electrocatalyst after tested in a prototype PEM flow-cell water electrolyzer with an applied current density of 100 mA cm^{-2}, operated at 60 °C. **(d-i)** EDX elemental mapping Mo and Te elements from the 1T'-$MoTe_2$ film grown on CC support, after electrolysis. EDX showing non-stoichiometry due to Te surface passivation.

References:

(1) Kang, Z. Y.; Yang, G. Q.; Mo, J. K.; Yu, S. L.; Cullen, D. A.; Retterer, S. T.; Toops, T. J.; Brady, M. P.; Bender, G.; Pivovar, B. S.; Green, J, B.; Zhang, F. Y. Developing titanium micro/nano porous layers on planar thin/tunable LGDLs for high-efficiency hydrogen production. *Int J Hydrogen Energ* **2018**, *43* (31), 14618-14628. DOI: 10.1016/j.ijhydene.2018.05.139.

(2) Lu, D.; Ren, X.; Ren, L.; Xue, W.; Liu, S.; Liu, Y.; Chen, Q.; Qi, X.; Zhong, J. Direct Vapor Deposition Growth of 1T′ MoTe2 on Carbon Cloth for Electrocatalytic Hydrogen Evolution. *Acs Appl Energ Mater* **2019**, *3* (4), 3212-3219. DOI: 10.1021/acsaem.9b01589.

(3) McCrory, C. C. L.; Jung, S. H.; Peters, J. C.; Jaramillo, T. F. Benchmarking Heterogeneous Electrocatalysts for the Oxygen Evolution Reaction. *J Am Chem Soc* **2013**, *135* (45), 16977-16987. DOI: 10.1021/ja407115p.

(4) Piñeiro García, A.; Perivoliotis, D.; Wu, X.; Gracia-Espino, E. Benchmarking Molybdenum-Based Materials as Cathode Electrocatalysts for Proton Exchange Membrane Water Electrolysis: Can These Compete with Pt? *Acs Sustain Chem Eng* **2023**, *11* (20), 7641-7654. DOI: 10.1021/acssuschemeng.2c07201.

(5) Xie, Z.; Yu, S.; Ma, X.; Li, K.; Ding, L.; Wang, W.; Cullen, D. A.; Meyer, H. M.; Yu, H.; Tong, J.; Wu, Z.; Zhang, F, Y. MoS2 nanosheet integrated electrodes with engineered 1T-2H phases and defects for efficient hydrogen production in practical PEM electrolysis. *Appl Catal B-Environ* **2022**, *313*. DOI: 10.1016/j.apcatb.2022.121458.
